# High-dose cerium dioxide nanorods induce behavioral and electroencephalographic abnormalities through 5-HT1B receptor signaling disruption with an evaluation of anpirtoline's therapeutic potential

**DOI:** 10.1016/j.mtbio.2026.103749

**Published:** 2026-10-14

**Authors:** Mingshan Liu, Yuedong Li, Chunhao Shen, Hongyi Huang, Quanlei Liu, Bin Fu, Bihan Wu, Xiaomin Wu, Yumin Luo, Yongzhi Shan, Xuegang Niu, Guoguang Zhao

**Affiliations:** a Department of Neurosurgery, Xuanwu Hospital of Capital Medical University, No. 45 Changchun Street, Xicheng District, Beijing, 100053, China; b Brain Research Innovation and Translation Laboratory, Xuanwu Hospital of Capital Medical University, No. 45 Changchun Street, Xicheng District, Beijing, 100053, China; c Clinical Research Center for Epilepsy, Capital Medical University, No. 45 Changchun Street, Xicheng District, Beijing, 100053, China; d National Medical Center for Neurological Diseases, No. 45 Changchun Street, Xicheng District, Beijing, 100053, China; e Department of Neurosurgery, Neurosurgery Research Institute, The First Affiliated Hospital, Fujian Medical University, No. 20, Chazhong Road, Taijiang District, Fuzhou, 350005, China

**Keywords:** Epileptic susceptibility, Electroencephalogram abnormalities, Cerium dioxide nanorods, Htr1b, Serotonin, Abnormal exploratory behaviors

## Abstract

Cerium dioxide nanorods (CeO_2_ NRs) have drawn extensive research attention in the past few years for their exceptional generation of reactive oxygen species (ROS) and intrinsic catalytic properties. The two ends of the biological effect show a dose-dependent difference: protection of cells at low doses and damage to cells at high doses. Although the above processes are well documented in other biological contexts, the neurotoxicity induced by acute exposure to CeO_2_ NRs, as well as its mechanism and possible prevention strategies, have yet to be elucidated. In the present study, we have found that CeO_2_ NRs at a concentration of 500 μg/mL or higher induced neuronal apoptosis and reduced cell viability in vitro. After intranasal instillation in mice, CeO_2_ NRs accumulated in the lysosomes and nuclei of neurons in the hippocampus and striatum. There was an increase in these abnormal exploratory behaviors, heightened seizure susceptibility, and altered electroencephalographic (EEG) oscillations. Integrated transcriptomic and proteomic profiling further indicated disruption of serotonin (5-HT) metabolism, caused by a downregulation of the 5-HT1B receptor, and thus provided a possible molecular mechanism for the behaviors and EEG abnormalities observed. Considerable changes also occurred in the various serotonergic mediators, neuronal excitability indicators, and inflammatory markers. Pharmacological activation of the 5-HT1B receptor with anpirtoline partially reversed the behaviors and EEG changes induced by CeO_2_ NRs.

## Highlights

• CeO_2_ NRs deposited in hippocampus and striatum after intranasal instillation.
• High-dose CeO_2_ NRs altered exploratory behavior, EEG, and seizure susceptibility.
• Multi-omics showed CeO_2_ NRs downregulated Htr1b and disrupted 5-HT metabolism.
• Anpirtoline partly rescued behavioral, EEG, and molecular abnormalities.

## Graphical abstract

**Figure:**
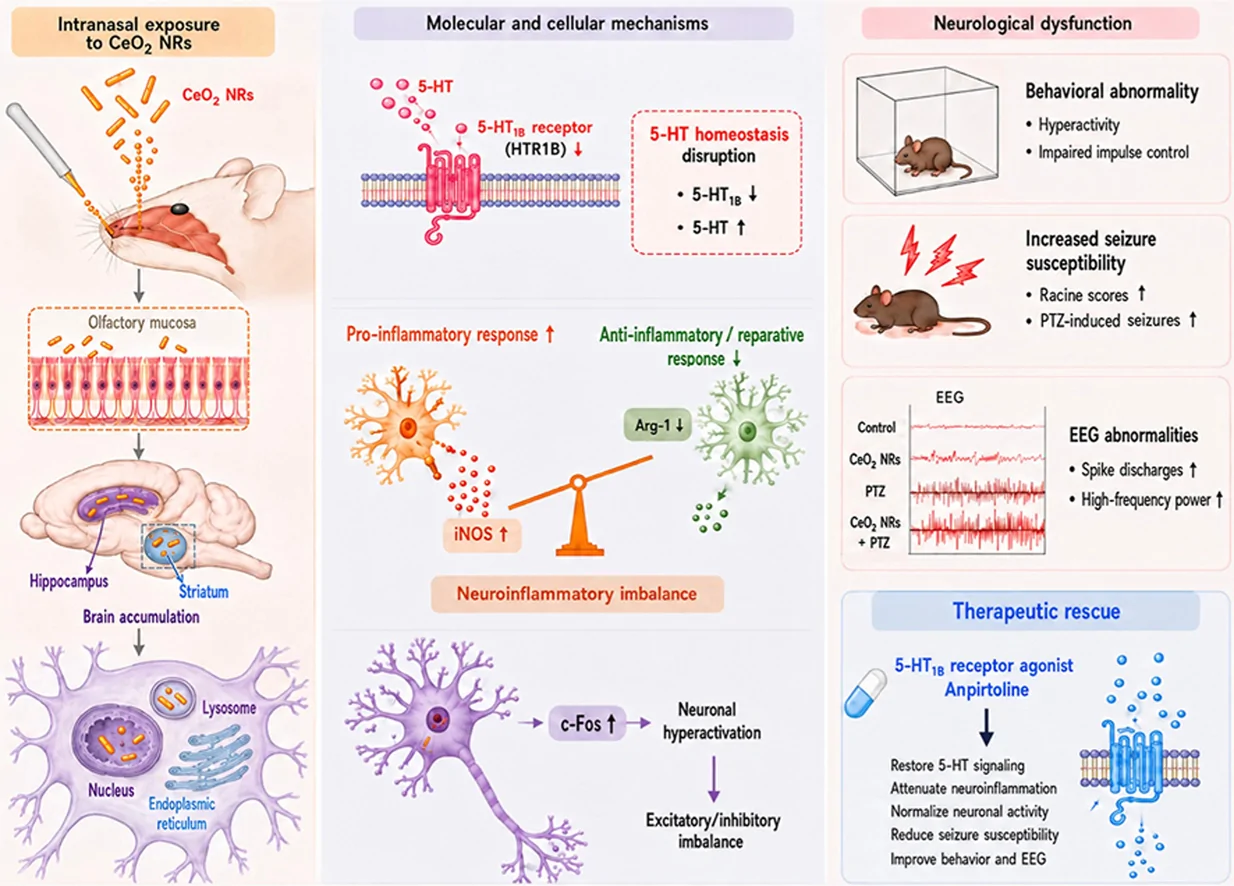

## 1 Introduction

Cerium Dioxide nanorods (CeO_2_ NRs) are a type of functional nanomaterials with redox properties due to the reversible conversion of Ce^3+^ and Ce^4+^ [1]. CeO_2_ NRs have redox cycling properties that can control the production of ROS and oxidative stress, so extensive research has been conducted on their antioxidant biomedical applications and disease treatment. [2]. Given the characteristics above, CeO_2_ NRs have a wide range of applications in many fields, including biomedicine [3], [4], catalysis [5], energy conversion [6], and environmental remediation [7]. With the increase in production and applications of CeO_2_ NRs, serious problems related to their potential release during the life cycle from manufacturing to disposal have also gradually been emerging [8], [9]. CeO_2_ NRs can enter the human body after being released in the environment by inhalation, oral ingestion or skin contact [10]. Inhaled or absorbed nanoparticles (NPs) can be transported in the blood to reach the central nervous system. Earlier studies have shown that the biological effects of CeO_2_ nanomaterials are generally dualistic. Since CeO_2_ NRs possess peroxidase-like activity [11], they can also be used as antioxidants to reduce reactive oxygen species (ROS) via a redox-related ROS-scavenger mechanism [12]. At the same time, changes in exposure time, exposure dose and particle morphology can also cause inflammation and damage to cells by CeO_2_ [13]. Therefore, a comprehensive evaluation of the potential toxicity and biological risks of CeO_2_ NRs has gradually attracted the attention of the nanomaterial safety community.

Some previous studies on animals have also shown that CeO_2_ NRs are distributed to various organs, including the liver, lungs, spleen and kidneys, after different exposure routes, and thus induce diverse inflammatory reactions, damage to histopathological structures and organ dysfunction [14]. CeO_2_ NRs did not generally cause obvious death after acute and subacute inhalation exposure. Alveolar macrophages may internalize them and thus cause a localized inflammatory response. In addition, the CeO_2_ may be carried by the blood to other parts of the body outside the lungs and cause systemic damage [15]. Based on the above observations, it can be concluded that the biosafety of CeO_2_ NRs should not be judged by acute toxicity or single-point cytotoxicity experiments alone; rather, their biodistribution and clearance characteristics are largely influenced by the duration of exposure [16]. Studies of inhalation exposure have reported that CeO_2_ NRs can reach extrapulmonary tissues, and cerium-associated signals have been detected in brain tissue after a single exposure [17].

Although many studies have been conducted on CeO_2_ nanomaterials in the peripheral organs of the body, such as the liver, lungs and kidneys, and the immune system, their effects on the central nervous system (CNS) have received relatively little attention. At present, studies have shown that the biological effects of CeO_2_ nanomaterials in the nervous system are complex and not uniformly beneficial or harmful [18]. On the one hand, due to their antioxidant and anti-inflammatory properties, CeO_2_ nanomaterials have been studied for brain injury [19]. At the same time, in vivo developmental studies have shown that CeO_2_ can be taken up by neural progenitor cells and affect neuronal and glial differentiation and growth, thus potentially impacting neurodevelopment [20]. Recently, in vivo experiments have started to explore the neurological effects of CeO_2_ exposure via inhalation or intraperitoneal injection [21]. Based on the above studies, CeO_2_ may induce oxidative damage to brain tissue, alter cholinergic metabolism, and cause pathological changes in the hippocampus. Most of the previous research has focused on traditional CeO_2_ and classic toxicological effects, such as oxidative stress-induced damage [22]. Whether CeO_2_ nanomaterials cause behaviors changes, neuronal hyperexcitability and neurotransmitter dysregulation after intranasal administration has not been determined.

To address the knowledge deficiencies mentioned above, a mouse model for intranasal delivery of CeO_2_ NRs was built to investigate their effects on neurological functions systematically. The CeO_2_ dose used in our study was selected with reference to previous toxicological studies on CeO_2_. One study reported that intranasal administration of CeO_2_ at 40 mg/kg (twice weekly for 3 weeks) induced oxidative brain damage and cognitive impairment [21]. Another study using the same dose of 40 mg/kg (intranasal instillation, once daily) found reduced chromatin in hippocampal cells [23]. In addition, a study using intratracheal instillation of CeO_2_ at 0.5 mg/kg demonstrated that CeO_2_ induced oxidative stress, inflammation, and DNA damage in multiple organs including the brain [24]. These findings suggest that the 40 mg/kg dose may be excessively high, directly triggering oxidative stress and neuronal cell death. In contrast, the objective of our study was to investigate whether intranasal administration of CeO_2_ NRs induces behavioral changes, neuronal hyperexcitability, and neurotransmitter alterations, which distinguishes our work from previous studies. Therefore, we selected a dose of 10.5 mg/kg for the subsequent in vivo experiments.

Behavioral tests and seizure susceptibility tests were first conducted to determine whether exposure to CeO_2_ NRs caused functional disorders, and then electroencephalogram (EEG) recordings were performed to observe changes in neuronal network activity and excitability (Fig. 1). Then, an abnormal accumulation of CeO_2_ was observed in the tissue, and cell death occurred at the cellular level. Integrated multi-omics analyses of transcriptomic (RNA-seq) and quantitative proteomic data were also performed to identify molecular changes induced by CeO_2_ NR exposure. The above results show that a reduction in the expression of the 5-hydroxytryptamine receptor 1 B (5-HT1B receptor) disrupted serotonergic transmission and is thus one of the molecular mechanisms underlying the behavioral and EEG changes observed. Based on the results of several omics experiments and neurological symptoms, we have focused on changes in the serotonergic system, inflammation and markers of neuronal hyperexcitability. Our study found that CeO_2_ NRs disrupt 5-hydroxytryptamine (5-HT) homeostasis in the hippocampus and striatum by inhibiting 5-HT1B receptor signaling, and are associated with a neuroinflammatory microenvironment and increased neuronal excitability. Thus, CeO_2_ NRs-exposed mice showed abnormal exploratory behaviors and EEG abnormalities. A pharmacological rescue experiment was set up to investigate whether supplementing serotonergic signals could alleviate the behavioral and electrophysiological disorders induced by CeO_2_ NRs, and the selective 5-HT1B receptor agonist anpirtoline (AP) was employed [25]. We explored the molecular reasons for CNS damage caused by CeO_2_ NRs and propose new anti-neurotoxic treatments in this study.

**Fig. 1:**
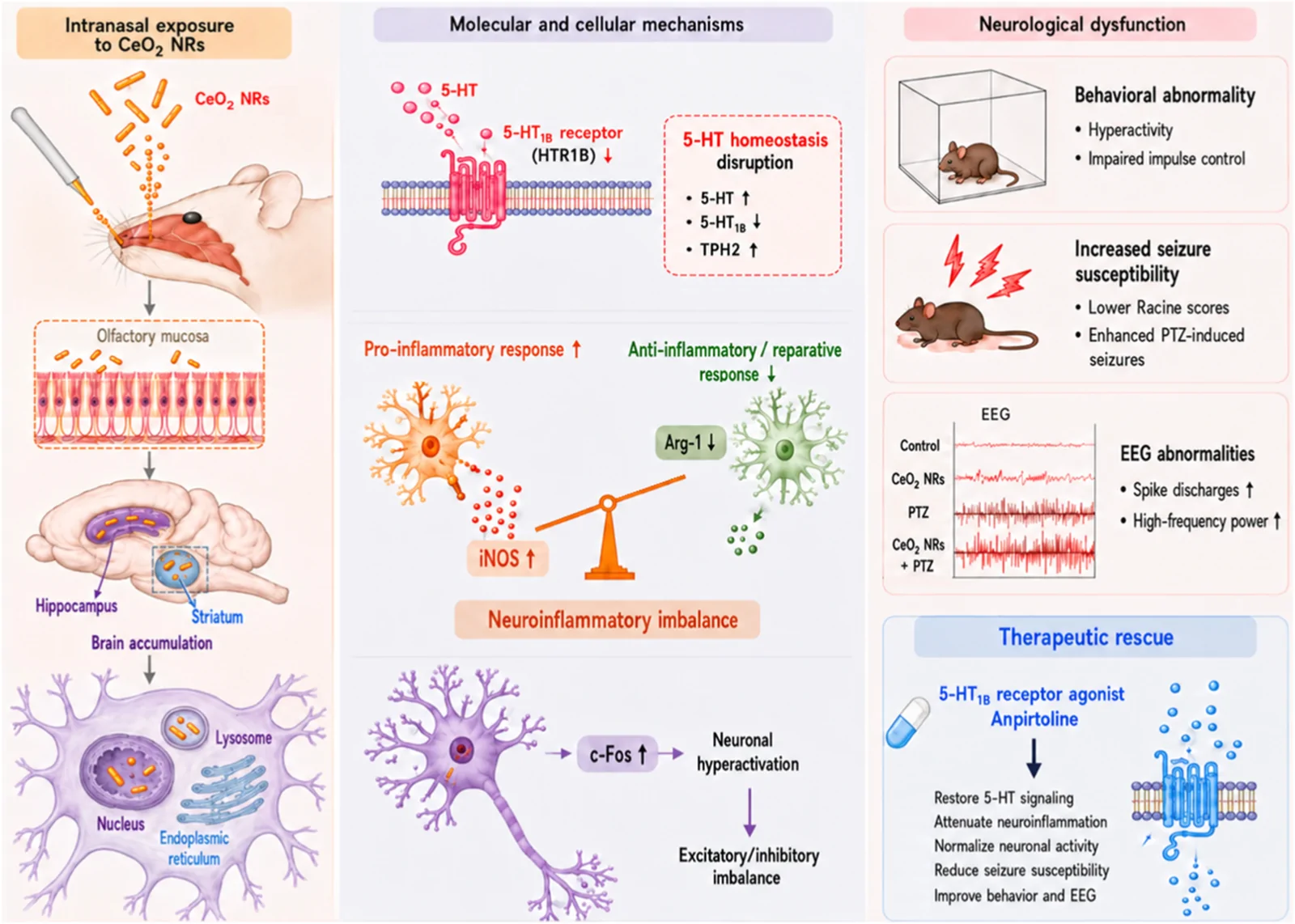
Schematic illustration of the study. Intranasally administered CeO_2_ NRs accumulate in the hippocampus and striatum and suppress 5-HT1B receptor expression. This suppression caused local inflammation and neuronal hyperexcitability, resulting in changes to the EEG and altered exploratory behaviors; thus, 5-HT1B receptor may be a potential therapeutic target.

## 2 Experimental section

### 2.1 Characterization of materials

Commercially available CeO_2_ NRs (103060, XFNANO, China) were selected for use in this study to ensure experimental consistency and reproducibility. Transmission electron microscopy (TEM; JEM-F200, JEOL, Japan) was used to observe the shape of CeO_2_ NRs. High-resolution transmission electron microscopy (HRTEM; JEM-F200, JEOL, Japan) was used to observe the crystal lattice structure of CeO_2_ NRs at a high resolution. Dispersion of CeO_2_ NRs in neuronal cells from brain tissue was examined using biological transmission electron microscopy (Bio-TEM; HT-7700, Hitachi, Japan). Energy-dispersive X-ray spectroscopy (EDS, XFlash, Bruker, Germany) was used to determine the elemental composition of CeO_2_ NRs. X-ray diffraction (XRD; D8 Advance, Bruker, Germany) was employed to analyze the structural modifications of CeO_2_ NRs before and after simulation of lysosome-mediated degradation in vitro. A zetasizer nano zs90 (Malvern, UK) was used to measure the size distribution of CeO_2_ NRs before and after simulation of lysosome-mediated degradation in vitro.

### **2.2** **Cytotoxicity of CeO**_2_**NRs to neuronal cells**

Primary neuronal extraction and culture. Cortical neurons were isolated from embryonic day 15 C57BL/6N mice (Beijing Vital River Laboratory Animal Technology Co., Ltd., Beijing, China). Under sterile conditions, the cerebral cortices were removed and mechanically cut into approximately 1 mm^3^ pieces. We added 0.25% trypsin (T1300, Solarbio, China) to dissociate the tissue fragments for 5 min, then gently triturate to obtain a single-cell suspension. Digestion was interrupted by adding Dulbecco's modified Eagle's medium (DMEM) containing 10% fetal bovine serum (FBS). Centrifuge the cell suspension at 1000 rpm for 5 min and resuspend it in DMEM containing 10% FBS. Dissociated neurons were added to six-well plates pre-coated with poly-L-lysine (PB180523, Procell, China). The next day, the plating medium was changed to Neurobasal medium (21103049, Thermo Fisher Scientific, USA) with 1% Penicillin-Streptomycin Liquid (P1400, Solarbio, China), 2% B-27 supplement (17504044, Thermo Fisher Scientific, USA), and 1% GlutaMAX supplement (35050061, Thermo Fisher Scientific, USA). Half of the culture medium was changed every three days, and neurons were kept in vitro for 7 days before the next experiment.

Cellular uptake of CeO_2_ NRs by neurons. To visualize cellular uptake of CeO_2_ NRs, CeO_2_ NRs were first labeled with rhodamine B (IR0280, Solarbio, China) by co-incubation at room temperature for 2 h, and dye conjugation occurred through electrostatic interaction. Rhodamine B-labeled CeO_2_ NRs were centrifuged at 8000 rpm for 10 min to obtain the pellet, and then three washes with sterile double-distilled water were performed to remove unbound dye. Fluorescently labeled NRs were added to the primary neuronal cultures at a concentration of 100 μg/mL for 1 h. After this incubation, the excess CeO_2_ NRs were washed away, and then the cells were used for immunofluorescence staining. Neurons were briefly incubated with the anti-microtubule-associated protein 2 (MAP2) primary antibody (ab5392, Abcam, UK) and then added to a Goat Anti-Chicken IgY H&L secondary antibody (ab150169, Abcam, UK). Nuclear counterstaining was done with 4′,6-diamidino-2-phenylindole (DAPI, ID2250, Solarbio, China). Dispose of the excess stain solution and then acquire fluorescence images with a fluorescence microscope.

Neuronal apoptosis assay. To determine whether apoptosis had occurred, primary neurons were treated with an increasing range of concentrations of CeO_2_ NRs (0, 100, 300, 500 and 1000 μg/mL) for 3 days. Then, using the instructions from the manufacturer, terminal deoxynucleotidyl transferase dUTP nick-end labeling (TUNEL) staining was performed using an apoptosis detection kit (C1089, Beyotime, China).

Cell viability assay. Parallel cultures were treated with the same series of concentrations of CeO_2_ NRs (0, 100, 300, 500, and 1000 μg/mL) for 3 days. Neuronal viability and cytotoxicity were tested with a Cell Viability and Cytotoxicity Assay Kit (C2015S, Beyotime, China) according to the instructions from the manufacturer, and live cells were green while dead cells appeared red.

### 2.3 CeO_2_ NRs-induced primary neuronal hyperexcitability assay

Primary neurons were isolated from embryonic day 15 mice, cultured for 7 days until maturation, and then treated with CeO_2_ NRs (500 μg/mL) for 3 days. Neurons were briefly incubated with the anti-MAP2 primary antibody. c-Fos was used as a marker of neuronal hyperexcitability. Subsequently, the neurons were imaged and analyzed by immunofluorescence.

### 2.4 Measurement of intracellular calcium levels in neurons

On the day of the experiment, the culture medium was removed, and the cells were gently washed twice with Ca^2+^/Mg^2+^-free Hank's balanced salt solution (HBSS). Then, the neurons were incubated with Fluo-4-AM (final concentration 5 μmol/L) in serum-free Neurobasal medium for 30 min in the dark. After incubation, the dye solution was discarded, and the cells were washed three times with HBSS, followed by an additional 20-min incubation at 37 °C in the dark to allow complete de-esterification of Fluo-4-AM. Fluorescence images were acquired using a confocal microscope (excitation 488 nm).

### 2.5 CeO_2_ NRs treatment of BV2 cells

In vitro, BV2 cells (a microglial cell line, CL-0493, Procell, China) were plated and cultured in DMEM medium supplemented with 10% fetal bovine serum (SA111.02 F, Cellmax, China) for 2 days at 37 °C in a 5% CO_2_ atmosphere. Subsequently, CeO_2_ NRs were added to the culture medium at a final concentration of 500 μg/mL. One day later, the BV2 cells were labeled with anti-ionized calcium-binding adaptor molecule 1 (Iba-1) and stained for inducible nitric oxide synthase (iNOS), followed by fluorescence imaging and quantitative analysis.

### 2.6 Animals and treatment

Eight-week-old male C57BL/6J mice were purchased from Beijing Vital River Laboratory Animal Technology Co., Ltd. (Beijing, China). Mice were kept in a 12-h light/dark cycle, maintained at 25 ± 2 °C and a relative humidity of 50-55%, with free access to standard diet and water. Mice were kept in the above environment for 2 days before the experiment.

The two sets of experiments in this work are independent. The two groups of animals were randomly assigned to the control group and the group exposed to CeO_2_ NRs in the first experiment. Control mice were given 40 μL of saline via intranasal instillation, and those in the exposure group received an equal volume of CeO_2_ NRs suspension at 10.5 mg/kg once daily for three consecutive days. After the last exposure, all of the mice were placed in a set of behaviors tests, seizure-susceptibility tests and EEG recorded. Brain samples were then taken for histological immunofluorescence staining, transcriptome sequencing and proteomics.

To confirm whether serotonergic signaling is involved in the neurological effects of CeO_2_ NRs, an additional group of animals was added for drug treatment. These mice were randomly assigned to four groups: the control, the CeO_2_ NRs-exposed, the AP-only group, and AP-treated (CeO_2_ NRs-AP). The exposure schedule for CeO_2_ NRs was the same as that of the first experiment. An AP intervention group of selective 5-HT1B receptor agonist AP (0.5 mg/kg) was established; 30 min after each CeO_2_ NRs dose, it was administered intraperitoneally, and this treatment was repeated throughout the 3 days of exposure. Control and CeO_2_ NRs-only animals received an equal volume intraperitoneal injection of saline.

### 2.7 Content of CeO_2_ NRs in different brain regions

The acute-exposure protocol followed the same steps as above. Anesthesia and euthanasia were performed with isoflurane (R510-22-10, RWD Life Science, China), and then specific brain regions were carefully dissected, and approximately 12 mg of tissue from each region was collected. Tissue samples were then subjected to microwave digestion under the following conditions: The temperature was increased to 120 °C in 5 min and held for 5 min; then, it was raised to 180 °C over the next 5 min and kept at 180 °C for 15-20 min. Acid evaporation and volume adjustment were performed first, and then the concentration of cerium in the digests was measured by inductively coupled plasma optical emission spectrometry (ICP-OES; 5110 ICP-OES, Agilent, USA).

### 2.8 Behavioral experiments

Open-field test (OFT). OFT was used to examine whether the mice exposed to CeO_2_ NRs were hypoactive and anxious. The apparatus was a square arena 1m by 1m, and the central zone in the middle is a 0.5m by 0.5m square. A single mouse was placed in the arena and free to move for 10 min. At that time, the Noldus EthoVision XT system was tracking the movement of the animal. The two main indices we used were the time spent in the central area and the number of entries into the central zone. Between all experimental trials, both the arena floor and side walls were thoroughly wiped with 75% ethanol to eliminate olfactory cues like pheromones left by the prior animal.

Novel object recognition (NOR). The NOR task is employed to evaluate recognition memory. It was an open room and had three stages: habituation, familiarization and testing. Mice were kept in the test room overnight prior to the experiment to reduce stress from the new environment. Familiarizations: Place the two identical cylinders in the chamber and allow each mouse to explore one for 10 min. Then mice went back to their home cages. Twenty-four hours later, we replaced one cylinder with a triangular object and put each mouse back in the chamber for another 10 min of exploration. EthoVision XT software recorded both video and motion data, and therefore the exploration time of new objects could be compared with that of familiar objects.

Elevated Plus Maze test (EPM). One day before testing, mice were habituated to the experimental room. On the test day, each mouse was placed on the central platform of the elevated plus maze with its head facing an open arm. The 5-min session was recorded and analyzed using the Noldus EthoVision XT video tracking system. The software produced trajectory plots and heat maps of the locomotor activity, and the time spent in the open arms was expressed as a percentage of the total test duration. Between subjects, the maze was wiped with ethanol to remove olfactory cues.

Forced swimming test (FST). Each mouse was individually placed into a transparent cylinder filled with water. The test session lasted 6 min. During the final 4 min of the session, the proportion of time spent immobile was scored, with immobility defined as the mouse making only minimal movements necessary to keep its head above water. The percentage of immobility time was calculated as (immobility duration/240 s) × 100%.

Three-chamber social interaction test. One day before the experiment, the mice were acclimatized to the behavioral testing room. In the social preference test, each subject mouse was placed in the central compartment of the three-chamber apparatus and allowed to move freely between an empty cage and a cage containing an unfamiliar conspecific for 10 min. The social preference index was calculated as: (exploration time in the stranger side/total exploration time in both sides) × 100%. In the subsequent social novelty preference test, the empty cage was replaced with a novel unfamiliar mouse, and the first stranger mouse then served as the familiar mouse. The subject mouse was again placed in the central compartment and allowed to explore freely for 10 min. The social novelty preference index was calculated as: (exploration time in the novel side/total exploration time in both sides) × 100%.

### 2.9 Pentylenetetrazol (PTZ)-induced seizure susceptibility experiment

PTZ Seizure Susceptibility Test. A cumulative PTZ-induced seizure model in mice was built to investigate the effect of exposure to CeO_2_ NRs on seizure susceptibility. PTZ (12.5 mg/kg) was injected intraperitoneally, and about 10 min later, the animal had a seizure. A total of four PTZ injections were carried out at regular intervals. Racine scoring was used to grade the severity of seizures, and the number of PTZ challenges needed to reach each seizure stage was recorded.

### 2.10 PTZ-induced acute epilepsy model and EEG recording

EEG recording and analysis of PTZ-induced seizure activity. To observe epileptiform EEG changes during an acute seizure in mice, stereotaxic surgery was performed under anesthesia to implant a single-channel tungsten recording electrode in the left hippocampal CA1 region (AP: −2.0 mm, ML: 1.5 mm, DV: −1.8 mm). After a one-week recovery period following electrode implantation, mice received a single intraperitoneal injection of PTZ (50 mg/kg), and continuous EEG signals were recorded for 30 min in freely moving mice using an EEG acquisition system (NSH0128, Neuracle, China). A sampling frequency of 2000 Hz was used. Neuracle HEEG software was employed for processing the acquired EEG data, generating an EEG heatmap, and plotting it. MATLAB's Brainstorm Toolbox was used for EEG analysis and statistics at the time.

### 2.11 Immunofluorescence

Brain tissues were cryosectioned at 10 μm, and then mounted on glass slides. Permeabilize the sections with 0.3% Triton X-100 for 15 min and block with 5% bovine serum albumin blocking solution for 1 h. Then, the sections were added to a primary antibody solution at 4 °C overnight. Incubate, then wash the sections three times in phosphate-buffered saline (PBS), and repeat this three times. The first antibodies in this study were: anti-serotonin antibody (ab6336, Abcam, UK), anti-tryptophan hydroxylase 2 (TPH2) antibody (ab184505, Abcam, UK), anti-5-HT1B receptor antibody (ASR-022, Alomone, Israel), anti-neuronal nuclei (NeuN) antibody (ab104224, Abcam, UK), anti-Fos proto-oncogene, AP-1 transcription factor subunit (c-Fos) antibody (ab222699, Abcam, UK), anti-Iba-1 antibody (ab283346, Abcam, UK), anti-iNOS antibody (ab178945, Abcam, UK), and Arginase-1 monoclonal antibody (Arg-1, 66129-1-Ig, Proteintech, China). Wash the samples and then, in a well, add the corresponding secondary antibody for 1 h at room temperature, followed by three washes in PBS (5 min each). The secondary antibodies used in this study are as follows: Goat anti-Rat IgG H&L (ab150157, Abcam, UK), Goat anti-Rabbit IgG H&L (ab150080, Abcam, UK), Goat anti-Rabbit IgG H&L (ab150077, Abcam, UK), Goat anti-Mouse IgG H&L (ab150115, Abcam, UK) and Goat anti-Mouse IgG H&L (ab150116, Abcam, UK). Acquire fluorescence images using a fluorescence microscope last.

### 2.12 Statistical analysis

All data are presented as means ± standard deviations (SD). GraphPad Prism 9.5 (San Diego, CA, USA) was employed for statistical analysis. Measure the density of protein bands and fluorescent signals in ImageJ software (NIH, Bethesda, MD, USA). Group differences were examined by one-way analysis of variance (ANOVA) followed by Bonferroni's post-hoc test, and the results of comparisons between two independent groups were shown using an independent samples *t*-test. The levels of statistical significance are set as: *p < 0.05, **p < 0.01, ***p < 0.001, and ****p < 0.0001. NS means not significantly different.

## 3 Results and discussion

### 3.1 Characterization of CeO_2_ NRs and their neurotoxicity in vitro

Morphological and structural analysis was carried out to determine the physicochemical properties and changes in biological properties of pristine CeO_2_ NRs and those treated with artificial lysosomal fluid (ALF). ALF treatment changed the shape and crystal structure of the original nanorods significantly; thus, lysosome-mimicking conditions likely induced biotransformation. TEM images show that the original CeO_2_ NRs are rod-shaped, and most of the size distribution is between 6.5 and 11.7 nm (Fig. 2A). HRTEM showed a lattice spacing of about 0.31 nm for the as-prepared CeO_2_ NRs (Fig. 2B), and EDS confirmed that cerium (Ce) and oxygen (O) were present in these CeO_2_ NRs (Fig. 2C). XRD analysis showed a reduction in the intensity of diffraction peaks after ALF treatment, suggesting lower crystallinity (Fig. 2D). Among the peaks, the (111) reflection showed a significant reduction; thus, it can be concluded that the surface structure had been modified after exposure to ALF [22]. We also observed an additional peak broadening of about 30 per cent in the ALF-treated samples, which we attribute to lattice distortion caused by Ce^4+^ to Ce^3+^ reduction and the generation of oxygen vacancies in the lysosomal environment [26]. Consistent with the XRD results, TEM images also show that the ALF-treated NRs are still in a rod-like shape but have become rougher and less distinct at the surface (Fig. 2E). Based on particle size analysis, the ALF-treated NRs showed a size distribution of 7.5-13.5 nm, comparable to that of the pristine nanorods (6.5-11.7 nm), with a slightly broader distribution (Fig. 2E). HRTEM analysis shows a change in lattice spacing of about 0.41 nm (Fig. S1) Together, they suggest lattice expansion after ALF treatment. Fig. 2F showed that CeO_2_ NRs were taken up by neurons. To examine the dose-dependent effects of primary neuron death, cells were exposed to 0, 100, 300, 500 and 1000 μg/mL of CeO_2_ NRs for 3 days. TUNEL staining showed that 100 μg/mL reduced apoptosis, and concentrations of 500 μg/mL or more significantly increased apoptosis (Fig. 2G–I). A pattern was also observed in the live/dead staining; 500 μg/mL and above caused a significant increase in neuronal death (Fig. 2H–J). Finally, ICP-OES was used to measure the amount of cerium in various areas of the brain after intranasal administration, and thus it could be determined whether the NRs had reached the brain. Both the hippocampus and the striatum had significantly higher cerium accumulation than other areas (*p* < 0.0001) (Fig. 2K). Bio-TEM analysis of hippocampal neurons showed rod-shaped or particulate CeO_2_ structures inside the neurons (white arrows), and CeO_2_ NRs were also found in lysosomes (red arrows) (Fig. 2L). As observed in vitro lysosomal simulation model, intracellular CeO_2_ NRs were partially degraded; however, the lysosomal degradation ability was relatively weak and mainly affected the surface of the CeO_2_ NRs. Excess intracellular CeO_2_ NRs were then observed in the nucleus (yellow arrows). In conjunction with the results of TUNEL and cell viability assays, the above data indicate that abnormal intracellular accumulation of CeO_2_ NRs may cause neuronal damage and apoptosis.

**Fig. 2:**
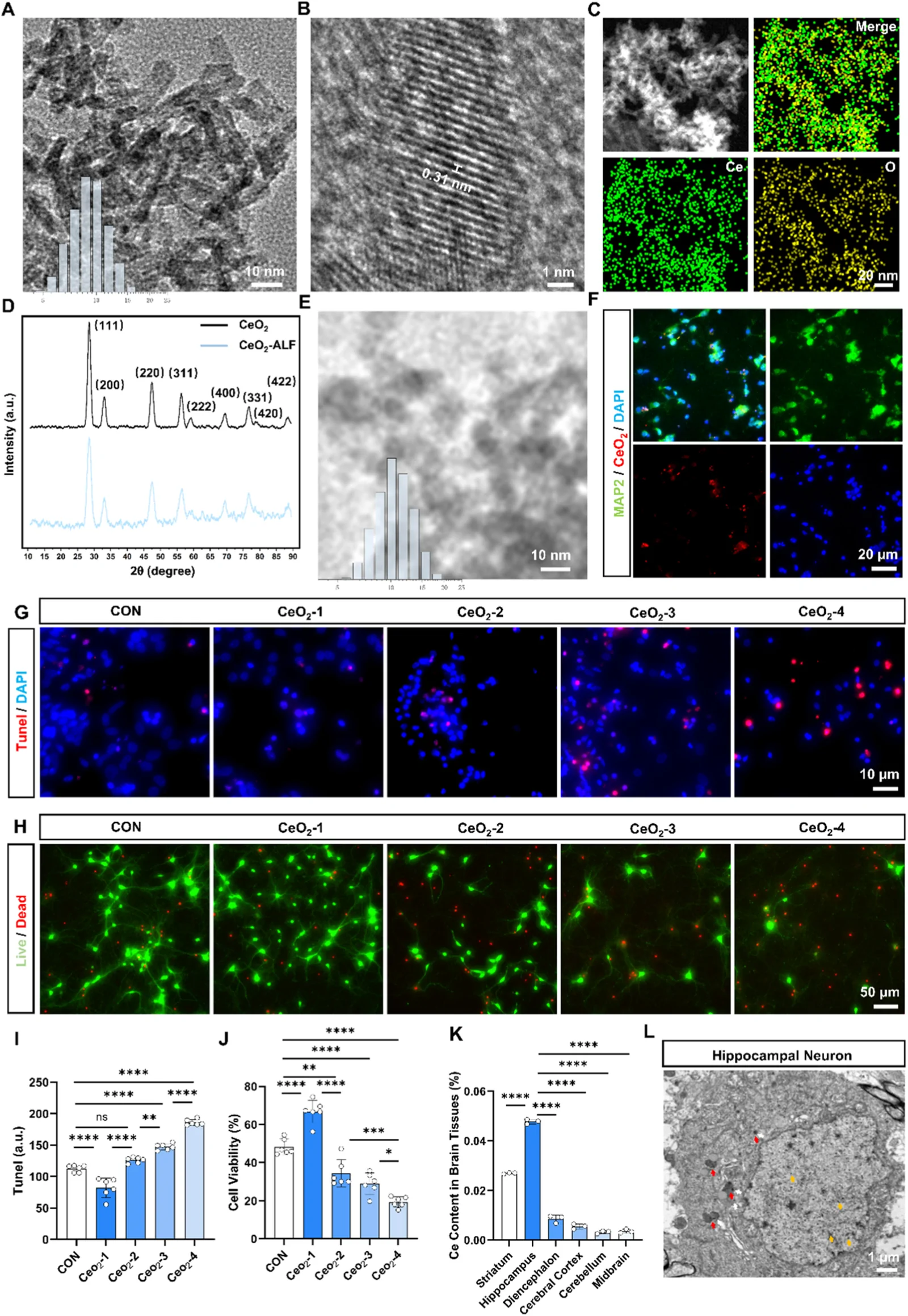
Characterization and neurotoxicity of CeO_2_ NRs in vitro. (A) TEM images and size distribution analysis of pristine CeO_2_ NRs. (B) HRTEM images of pristine CeO_2_ NRs. (C) EDS mapping of CeO_2_ NRs. (D) XRD patterns of CeO_2_ NRs before and after ALF treatment. (E) TEM images and size distribution analysis of ALF-treated CeO_2_ NRs. (F) Immunofluorescence images showing the internalization of CeO_2_ NRs by neurons. (G) TUNEL staining of neuronal apoptosis. (H) Live/dead staining of neurons. (I) Quantification of mean fluorescence intensity from TUNEL staining (n = 6). (J) Quantitative analysis of neuronal viability (n = 6). (K) Cerium concentrations in different brain regions measured by ICP-OES (n = 3). (L) Bio-TEM images of hippocampal neurons containing CeO_2_ NRs. White arrows are CeO_2_ NRs; red arrows are lysosomes; and yellow arrows show nuclear localization of CeO_2_ NRs. (For interpretation of the references to colour in this figure legend, the reader is referred to the Web version of this article.)

### 3.2 CeO_2_ NRs exposure induces behavioral abnormalities and EEG changes in mice

The behaviors and cognition of mice were tested in the OFT and NOR after exposure to CeO_2_ NRs. Trajectory tracking and heatmap analysis of the OFT showed that mice exposed to CeO_2_ NRs exhibited more activity in the central area of the arena than the control group (Fig. 3A). Quantitative analysis also showed that these mice spent a significantly longer time in the central area and made more frequent entries there (*p* < 0.01) (Fig. 3B and C). Exposed mice showed an increased approach behaviors to the novel object in the NOR test, as shown by longer exploration times and higher exploration frequencies compared with controls (*p* < 0.0001) (Fig. 3D and E). Collectively, the above results indicate that CeO_2_ NRs exposure alters exploratory behavior in an open environment, increases novelty-seeking tendencies, and impairs behavioral inhibition.

**Fig. 3:**
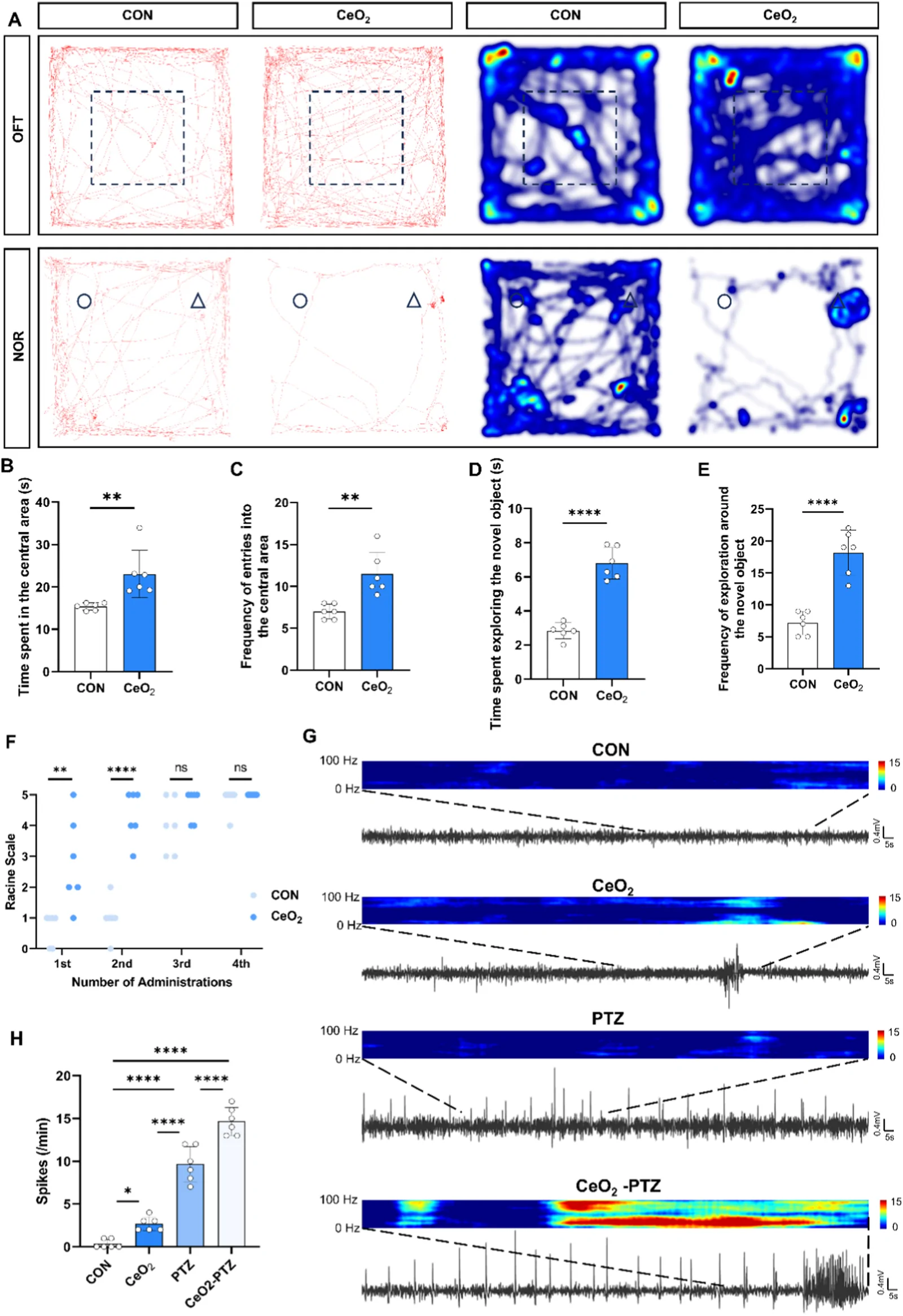
CeO_2_ NRs exposure induces behavioral abnormalities and EEG changes in mice. (A) Representative movement trajectories and heatmaps from the OFT and NOR. (B, C) Quantitative analysis of central zone exploration in the OFT (n = 6). (D, E) Quantitative analysis of exploration behaviors towards the novel object in the NOR (n = 6). (F) Quantitative analysis of seizure susceptibility (n = 6). (G, H) Representative EEG recordings and quantitative analysis in mice (n = 6).

Next, a cumulative PTZ administration model was used to determine whether exposure to CeO_2_ NRs increased seizure susceptibility induced by PTZ, and neuronal network excitability and seizure vulnerability were also investigated. Mice received intraperitoneal injections of PTZ (12.5 mg/kg) every 10 min, and after each injection, seizure-like behaviors was scored according to the Racine scale (Fig. 3F) [27], [28]. After the first PTZ injection, CeO_2_ NRs-exposed mice had Racine scores of 1-5, and the control group mostly remained at stages 0 and 1 (*p* < 0.01). After the second injection, the exposed mice showed more severe seizures, and the scores were mainly in stages 3 and 5; controls were mostly at stages 0-2, and stage 1 was the most frequent (*p* < 0.0001). As the cumulative PTZ dose increased, both groups showed a gradual worsening of seizure severity, and no significant difference in Racine scores was found between the two groups after the third and fourth injections (*p* > 0.05). According to the above data, CeO_2_ NRs-exposed mice reached a higher stage of seizures earlier under the same PTZ challenge; thus, CeO_2_ NRs exposure appears to reduce resistance to epileptogenic stimuli and increase seizure susceptibility.

To further verify the effect of neuronal network excitability caused by CeO_2_ NRs exposure, EEG recordings were taken from four groups of mice: control mice, CeO_2_ NRs-exposed mice, PTZ-treated mice, and CeO_2_ NRs-PTZ-treated mice (Fig. 3G). Representative time-frequency maps and raw EEG traces showed that control mice had relatively stable EEG activity and a low-power background signal. CeO_2_ NRs-exposed mice exhibited abnormal burst-like discharges and occasional epileptiform spikes, as well as local increases in time-frequency power. PTZ-treated mice had frequent epileptiform spikes in the EEG. Mice exposed to CeO_2_ NRs were then subjected to a PTZ challenge; these mice showed more frequent and prolonged epileptic discharges, prominent burst activity, and an increase in spectral power. Quantitative analysis of epileptiform spike frequency showed that CeO_2_ NRs-exposed mice had a higher spike frequency than controls (*p* < 0.05). PTZ-treated mice showed an increase in spike frequency compared with both the control and CeO_2_ NRs-exposed groups (*p* < 0.0001) (Fig. 3H). In addition, the CeO_2_ NRs-PTZ group also showed a higher spike frequency compared with the PTZ group (*p* < 0.0001). Therefore, CeO_2_ NRs do not show overt seizure behavior but are sufficient to generate abnormal epileptiform discharges. Exposure to CeO_2_ NRs also enhances PTZ-induced epileptiform activity and is consistent with the increased seizure susceptibility in the cumulative PTZ challenge model.

It should be noted that the CeO_2_ concentration employed in the in vitro assays (up to 1000 μg/mL) exceeds the actual in vivo exposure levels. This dose range is conventionally used in cytotoxicity screening to establish the concentration-effect relationship and to evaluate neural cell performance. Nevertheless, the in vivo milieu is inherently complex and subject to various factors, such as administration route and blood-brain barrier permeability. Accordingly, extrapolation of in vitro cellular findings to in vivo physiological conditions is not warranted.

### 3.3 Integrated multi-omics analysis reveals CeO_2_ NRs-induced serotonergic dysregulation

To investigate the molecular changes in behaviors and EEG abnormalities induced by CeO_2_ NRs, combined transcriptomics and proteomics were employed. RNA was extracted from the brain tissue of CeO_2_ NRs-exposed mice and control mice, and then RNA-seq was performed to discover genome-wide changes in gene expression. Based on the observed changes in exploratory behaviors and EEG activity, we selected differentially expressed genes associated with neurotransmitter metabolism and inflammation for heatmap analysis (Fig. 4A). The volcano plot of differentially expressed genes showed that 33 genes were upregulated and 39 genes were downregulated in CeO_2_ NRs-exposed mice compared with controls (*p* < 0.05 and |log_2_FC| > 0.5) (Fig. 4B). Among the differentially expressed genes, several genes related to 5-HT and glutamine (Gln) metabolism were also significantly altered. CeO_2_ NRs exposure significantly decreased *Htr1b* expression. The 5-HT1B receptor gene codes for a neurotransmitter release regulator that also regulates serotonin and glutamate; therefore, its reduction may be responsible for the behavioral abnormalities and altered neural activity in the exposed mice (Fig. 4D) [29], [30]. The 5 HT1B receptor serves as both a presynaptic autoreceptor and a heteroreceptor, and through its role as an inhibitory G protein coupled receptor, it suppresses the release of multiple neurotransmitters [31]. These phenotypes are highly consistent with our observations in CeO_2_ NRs-exposed mice, which showed increased central zone activity in the OFT and prolonged exploration time of novel objects in the NOR test. Glutamate-ammonia ligase (*Glul*) is the gene encoding for glutamine synthetase; it converts glutamate into glutamine and maintains the glutamate-glutamine cycle in the central nervous system (CNS) [32]. In CeO_2_ NRs-treated animals, *Glul* levels were significantly reduced; thus, glutamine synthesis may have been inhibited (Fig. 4D). [33]. Gene Ontology (GO) analysis of the differentially expressed genes shows that there is strong enrichment of biological processes related to external stimulus response and aggressive behavior (Fig. 4C). Based on the above observations, CeO_2_ NRs affect behavior through alterations in serotonergic signaling, notably by reducing the expression of the 5-HT1B receptor. Gene set enrichment analysis (GSEA) was also performed on the results to determine which Kyoto Encyclopedia of Genes and Genomes (KEGG) pathways were affected, and it was found that oxidative phosphorylation, arachidonic acid-related inflammatory pathways, chemokine signaling and mitogen-activated protein kinase (MAPK) signaling were significantly enriched (Fig. 4E).

**Fig. 4:**
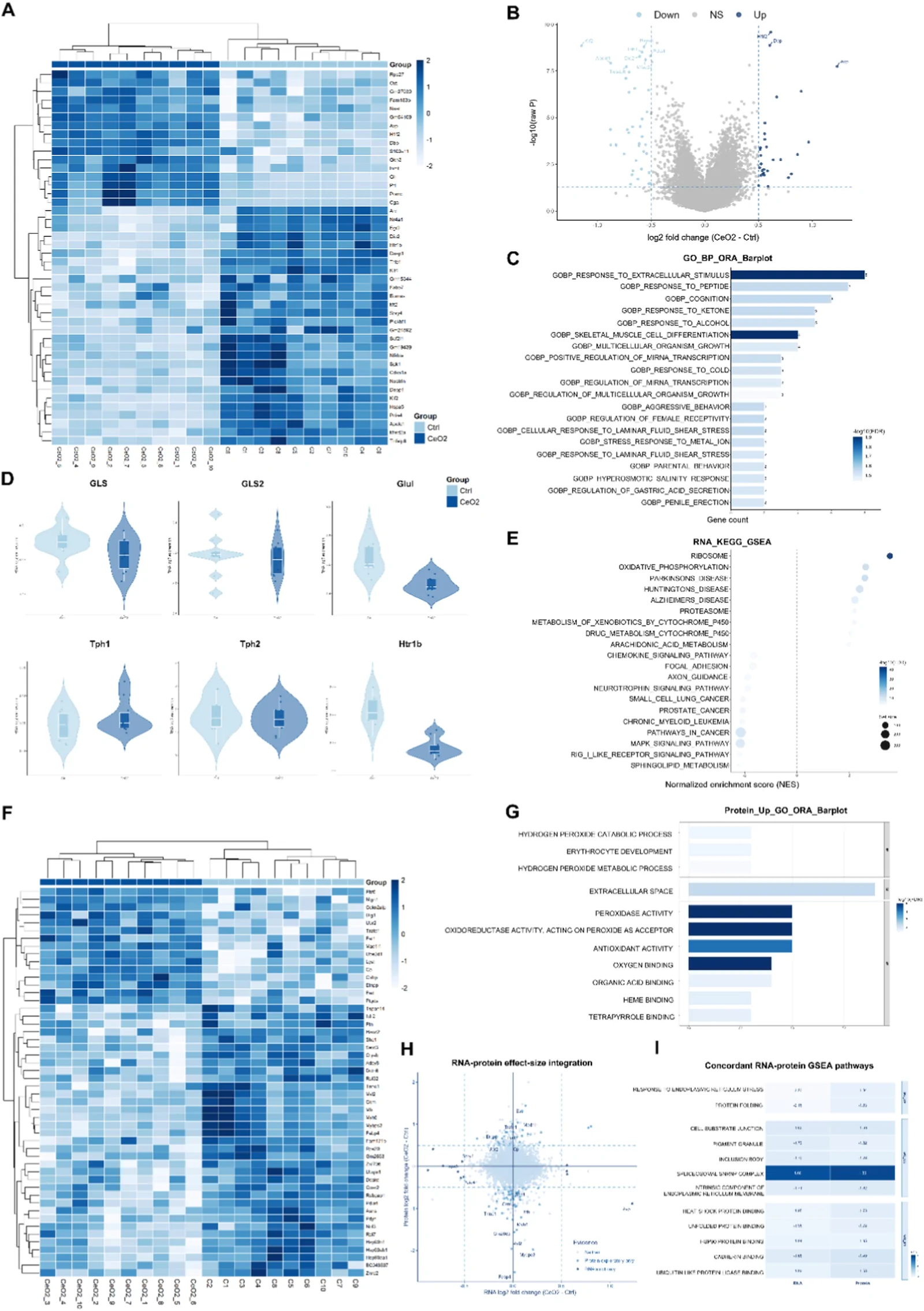
Transcriptomic and proteomic analyses of CeO_2_ NRs-exposed mice. (A) RNA-seq-based heatmap of gene expression changes. (B) Volcano plot of significantly altered transcripts. (C) GO terms enriched in biological processes related to differentially expressed genes. (D) Differential expression analysis of genes associated with 5-HT and Gln metabolism. (E) KEGG pathway enrichment analysis based on RNA-seq data. (F) Heatmap of differentially expressed proteins identified by quantitative proteomics. (G) GO enrichment bar plot of upregulated proteins. (H) RNA-protein effect-size integration analysis. (I) Concordant RNA-protein GSEA pathways.

Next, quantitative proteomics was used to obtain a full list of altered proteins in the brain after exposure to CeO_2_ NRs. Across 20 samples, a total of 6733 proteins were identified; among them, 43 were upregulated and 68 were downregulated (*p* < 0.05 and |log_2_FC| ≥ 0.5). A heatmap of the above differentially expressed proteins is shown in Fig. 4F. Among the downregulated proteins, *Kcnt2* [34] and *Slc24a3* [35] were found; both help to maintain the electrical stability of neurons. *Naa16* (a regulator of apoptosis) and *Ankrd44* (involved in stress response and repair) were also decreased following treatment with CeO_2_ NRs. All of the above changes suggest that CeO_2_ can disrupt neuronal homeostasis at the molecular level [36]. GO enrichment analysis showed that the differentially expressed proteins were mainly enriched in peroxide-related processes (Fig. 4G).The four-quadrant plot of RNA-protein effect-size integration shows that multiple genes exhibit concordant downregulation at both the RNA and protein levels. Among them, *Fabp4*, *Shc1*, *Myl2*, *Mybpc3*, and *Cp* are directly associated with oxidative stress, and *Fabp4* is additionally linked to 5-HT metabolism, regulated via the 5-HT/PPARγ pathway (Fig. 4H). However, these changes were modest and concordant, possibly due to non-matched animals for RNA and protein measurements, where inter-individual variability may have weakened the coupling. Still, the consistent trend in the integration analysis remains indicative. The integrated GSEA plot shows concordantly enriched pathways (FDR<0.05) at RNA and protein levels upon CeO_2_ treatment (Fig. 4I). Most pathways, including endoplasmic reticulum stress, protein folding, and heat shock protein (HSP) binding (all linked to proteostasis) were consistently downregulated. Therefore, CeO_2_ NRs likely interfere with the oxidative metabolic pathway in the brain and have induced damage to cells due to enhanced oxidative stress.

### 3.4 Exposure of CeO_2_ NRs induces serotonergic dysregulation, neuroinflammatory imbalance and neuronal hyperactivation in the hippocampus and striatum

To explore the molecular changes in behaviors abnormalities and increased seizure susceptibility induced by CeO_2_ NRs, immunofluorescence staining was employed to examine serotonergic signaling, inflammatory response and neuronal activation markers. According to the results of ICP-OES, cerium shows a relatively high accumulation in the hippocampus and striatum, so these two brain areas were selected for further molecular analysis. Changes in serotonergic markers occurred in both the hippocampus and the striatum after exposure to CeO_2_ NRs (Fig. 5A–C and Fig. S2A–E). CeO_2_ NRs-exposed mice showed an increase in 5-HT immunofluorescence intensity and a decrease in 5-HT1B receptor expression compared with control mice (Fig. 5B–D and Fig. S2D and E). No significant difference in TPH2 fluorescence intensity was found among the experimental groups either (Fig. 5F and Fig. S2B). TPH2 is the rate-limiting enzyme of central 5-HT synthesis, but its expression was not altered by exposure to CeO_2_ NRs. It can be inferred from the above data that the increase in local 5-HT concentration is not due to increased production; rather, it may be the result of modified utilization, secretion or feedback regulation of serotonin. At the same time, the 5-HT1B receptor, which is responsible for presynaptic inhibition and feedback control of neurotransmitter release, was downregulated; thus, there was a loss of inhibitory serotonergic modulation. All of the above show that CeO_2_ NRs disrupt serotonergic homeostasis in both the hippocampus and striatum. Next, c-Fos, a typical immediate-early gene and marker of neuronal excitation, was studied [37]. CeO_2_ NRs-treated mice showed a significant increase in c-Fos fluorescence intensity in the hippocampus compared with the control group (Fig. 5E and G), which is consistent with enhanced neuronal activity and increased network excitability. Moreover, CeO_2_ NRs treatment led to increased c-Fos expression and elevated intracellular Ca^2+^ levels in primary neurons, both of which clearly demonstrate that the neurons were directly provoked into aberrant hyperexcitability (Fig S3A, B and Fig S4A, B). Inflammatory markers in the hippocampus were also measured. CeO_2_ NRs-exposed mice had stronger iNOS fluorescence and weaker Arg-1 fluorescence compared with control mice (Fig. 5H–K). Higher iNOS indicates an increased local pro-inflammatory response and nitrosative stress, and a lower Arg-1 suggests impaired immune regulation and tissue repair. The mutual regulation of iNOS and Arg-1 suggests that CeO_2_ NRs promote an inflammatory state in the hippocampus. The same changes occurred in the striatum (Fig. S5A–D), indicating that these CeO_2_ NRs induce inflammatory microenvironmental changes in multiple brain regions. Moreover, a marked increase in iNOS expression was observed in BV2 cells following CeO_2_ NRs treatment, demonstrating that CeO_2_ NRs are capable of directly driving microglial polarization towards a pro-inflammatory state (Fig. S6A and B). Based on the previous studies, we believe that the neuroinflammatory changes observed may be due to multiple mechanisms, such as serotonergic dysregulation caused by 5-HT1B receptor downregulation and inflammatory reactions associated with CeO_2_ accumulation. Additionally, given that TPH2 expression was not altered, the possibility of increased 5-HT synthesis can be excluded. This finding further supports that the 5-HT elevation is attributable to dysregulated release rather than enhanced synthesis. CeO_2_ NRs exposure led to Htr1b downregulation and reduced 5-HT1B receptor expression, which attenuated presynaptic inhibition and caused dysregulation of 5-HT release. This, in turn, induced neuronal hyperexcitability and microglial activation. Ultimately, these molecular events resulted in impaired behavioral inhibition and neuroinflammation, manifesting as abnormal exploratory behaviors and EEG abnormalities in mice. In short, the above results show that CeO_2_ NRs have multiple effects on the nervous system and affect various areas of the brain; thus, the existing concept of CeO_2_-induced neurotoxicity as only inflammation is insufficient.

**Fig. 5:**
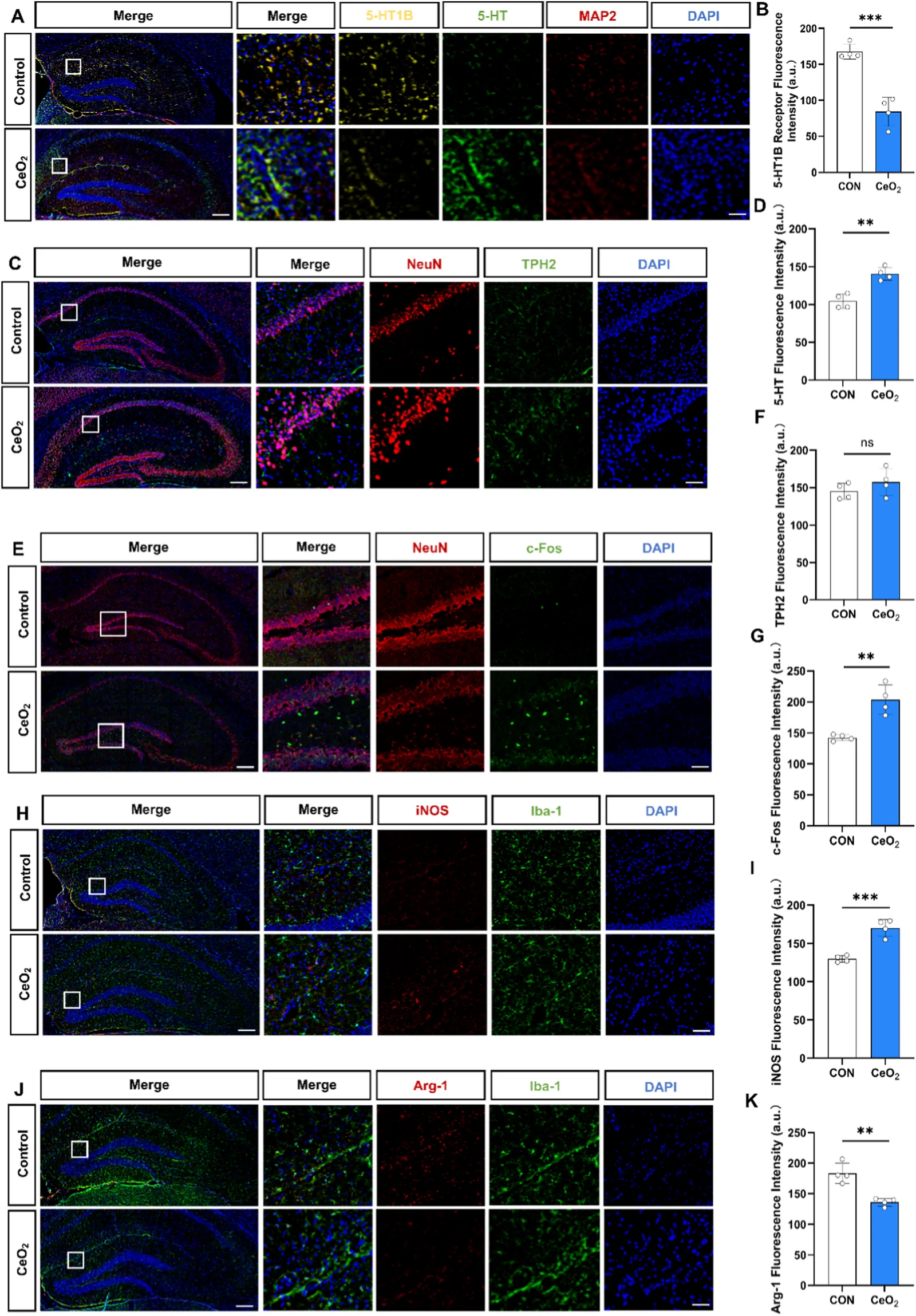
CeO_2_ NRs exposure induces serotonergic dysregulation, neuroinflammation and neuronal hyperactivation in the hippocampus and striatum (A, B, D). Immunostaining and quantification of 5-HT1B receptors and 5-HT are shown below. Scale bar: 100 μm. Scale bar for enlarged images: 25 μm (n = 4). (C, F) Immunofluorescence staining and quantitative analysis of TPH2. Scale Bar: 100 μm. Scale bar for enlarged images: 25 μm (n = 4). (E, G) c-Fos immunostaining and quantification. Scale bar: 100 μm Scale bar for enlarged images: 30 μm (n = 4). (H-K) Immunofluorescence staining and quantitative analysis of iNOS and Arg-1. Scale bar: 100 μm Scale bar for enlarged images: 25 μm (n = 4).

### 3.5 Activation of the 5-HT1B receptor by AP ameliorates CeO_2_ NRs-induced behavioral and EEG abnormalities

Based on the previous study, CeO_2_ NRs impair behavioral inhibition, alter recognition behaviors and increase the susceptibility of mice to PTZ-induced seizures. At the same time, transcriptomics and proteomics indicated that the disruption of 5-HT1B receptor signaling may be responsible for these neurological symptoms. To further determine whether impaired 5-HT1B receptor signaling contributes to CeO_2_ NRs-induced dysfunction and to explore a potential pharmacological intervention, AP, a selective 5-HT1B receptor agonist, was used to treat mice, and its effects on behavior and seizure susceptibility were evaluated. CeO_2_ NRs-exposed mice in the OFT entered the central zone more often and spent more time there than the control group (Fig. 6A–D, E). AP administration partially reversed the behaviors change, and the level of activity in the central zone was reduced to that of control mice. In the novel object recognition test of the experiment, mice exposed to CeO_2_ NRs showed abnormal exploration behavior for new objects, with longer and more frequent exploration times compared with the control group. AP-treated mice showed reduced novel object exploration compared with the CeO_2_ NRs-exposed group (Fig. 6B–F, G). Fig. 6C presents the representative track plots and heat maps of the EPM. There was no difference in open-arm exploration time between the AP and CON groups (Fig. 6H). The CeO_2_ group spent a significantly greater proportion of time in the open arms than all other groups, while the CeO_2_-AP group showed a modestly higher open-arm time than the CON group. This suggests that CeO_2_ promoted risk-taking exploration, and AP partially reversed this effect. In the FST, the CeO_2_ group exhibited an immobility time percentage of approximately 20%, significantly lower than that of the control and AP groups (about 60% each). In contrast, the CeO_2_-AP group displayed a partial restoration of immobility time, with values intermediate between the CeO_2_ and control groups (Fig. 6I). In the three-chamber social test, both indices in the CeO_2_ group were significantly higher than those in all other groups, demonstrating that CeO_2_ exposure led to abnormally enhanced exploratory behavior. In contrast, the CeO_2_-AP group showed an amelioration of this abnormal exploratory behavior (Fig. 6J and K). Additionally, no significant difference was observed between the CON and AP groups, suggesting that AP treatment does not affect exploratory behavior in normal mice. This is presumably because the 5-HT metabolism in normal mice is maintained at a physiological level and is not influenced by AP administration.

**Fig. 6:**
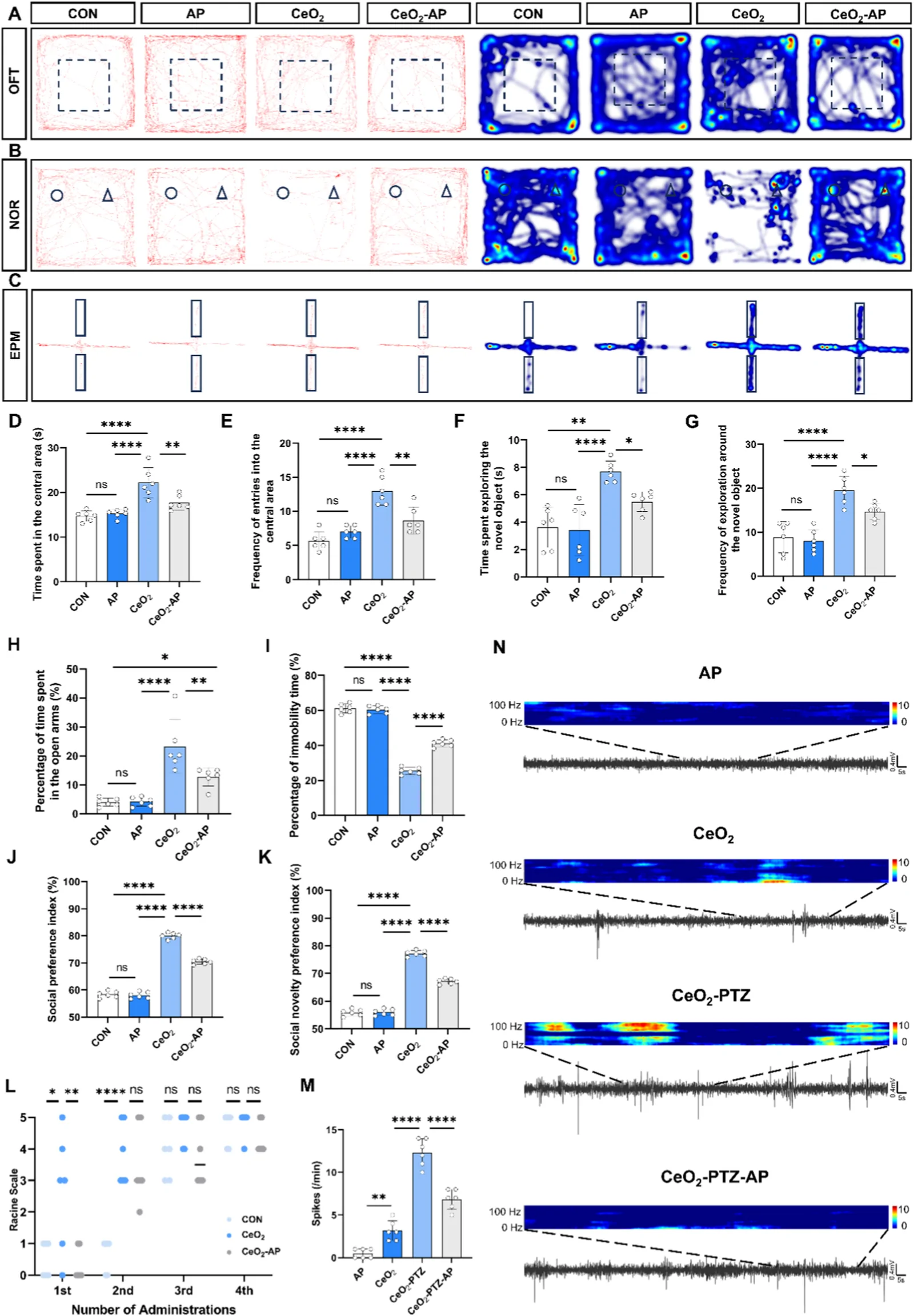
AP alleviates behavioral abnormalities and EEG alterations in CeO_2_ NRs mice. (A-C) Representative movement trajectories and heatmaps from the OFT, NOR and EPM. The triangle represents a new object. The rectangle represents the open arm. (D, E) Quantitative analysis of central zone exploration in the OFT (n = 6). (F, G) Quantitative analysis of novel object exploration in the NOR (n = 6). (H) Percentage of time spent in open arms (n = 6). (I) Percentage of immobility time in the forced swimming test (n = 6). (J) Social preference index in the three-chamber test (n = 6). (K) Social novelty preference index in the three-chamber test (n = 6). (L) Quantitative analysis of seizure susceptibility (n = 6). (M, N) Representative EEG traces and quantitative analysis (n = 6).

Therefore, it can be concluded that 5-HT1B receptor activation partially reverses the abnormal exploratory behaviors induced by CeO_2_ NRs exposure. In the cumulative PTZ challenge test, AP-treated mice had a lower Racine score in the early stage of PTZ stimulation compared to CeO_2_ NRs-exposed mice (Fig. 6L). After the first PTZ injection, the distribution of Racine scores in the CeO_2_-AP group was the same as in the control group. After the second injection, AP-treated mice still showed some seizure-like behavior, but fewer animals reached higher stages of seizures than those exposed to CeO_2_ NRs. After the third and fourth injections, there were no significant differences in Racine scores among the three groups. Collectively, the above results show that pharmacological activation of the 5-HT1B receptor can reduce the increased seizure susceptibility induced by CeO_2_ NRs exposure.

Continuous EEG recordings were performed on CeO_2_ NRs-exposed mice to examine the effects of exposure to CeO_2_ NRs on neuronal network excitability and whether AP could alleviate these changes (Fig. 6M and N). Compared with the EEG baseline of the CON group in the preceding experiment (Fig. 3G and H), the EEG frequency in the AP group showed no significant deviation from that baseline, suggesting that AP administration per se does not affect neuronal excitability. Compared with CeO_2_ NRs-exposed animals that did not receive PTZ, those that received both CeO_2_ NRs and PTZ challenge exhibited more pronounced high-amplitude abnormal discharges and showed increased localized spectral power in time-frequency analysis. Therefore, PTZ stimulation is likely to increase the network hyperexcitability of CeO_2_ NRs-treated mice. AP treatment reduced abnormal EEG discharges and decreased high-power spectral regions compared with the CeO_2_ NRs-PTZ group. In short, the above results show that pharmacological activation of the 5-HT1B receptor reduces PTZ-induced hyperexcitability in neuronal networks of CeO_2_ NRs-exposed mice.

### 3.6 Activation of the 5-HT1B receptor ameliorates CeO_2_ NRs-induced serotonergic dysregulation, neuroinflammation and neuronal hyperactivation

To determine whether the activation of 5-HT1B receptors inhibits the molecular changes caused by CeO_2_ NRs, we performed western blot analysis to quantify 5-HT1B receptor protein levels in the hippocampus. In addition, immunofluorescence staining for serotonergic markers, inflammatory indicators and neuronal activation-related proteins was carried out in the hippocampus and striatum after AP treatment. For serotonergic signaling, CeO_2_ NRs-exposed mice showed an increase in 5-HT fluorescence and a reduction in 5-HT1B receptor expression in the hippocampus compared with controls, and AP treatment reversed the above trends (Fig. 7A–D). After normalization to an internal control to correct for batch-to-batch variations in protein loading. Western blot analysis revealed that no significant difference in 5-HT1B receptor protein levels was observed between the CON and AP groups. In contrast, the CeO_2_ group showed a pronounced downregulation of receptor expression. Importantly, the CeO_2_-AP group exhibited a partial restoration of 5-HT1B receptor protein levels (Fig. S7A and B). 5-HT levels dropped and 5-HT1B receptor expression rose in both brain areas, moving towards a control-like pattern. Pharmacological activation of 5-HT1B receptor thus partially corrected the serotonergic dysregulation caused by CeO_2_ NRs. A similar restorative effect was observed in the striatum (Figs. S8 and S9). Next, c-Fos was used to find the active neurons. CeO_2_ NRs increased c-Fos fluorescence in the hippocampus, while AP significantly reduced this signal (Fig. 7E and F), and thus 5-HT1B receptor activation suppressed neuronal hyperexcitability. CeO_2_ NRs exposure induced a pro-inflammatory state in the hippocampus and increased iNOS and reduced Arg-1 fluorescence compared to the control group (Fig. 7G–I). AP treatment increased Arg-1 compared with the CeO_2_ NRs group, indicating an alleviation of the inflammatory imbalance. The striatum also showed the same improvement after AP administration (Fig. S10) Thus, the restoration of 5-HT1B receptor signaling may reduce inflammation associated with serotonergic dysfunction. The above results indicate that AP can partially reverse the serotonergic abnormality, neuroinflammatory imbalance and neuronal hyperactivation induced by CeO_2_ NRs. Thus, impaired 5-HT1B receptor signaling is likely one of the reasons for CeO_2_ NR-mediated neurological disorders.

**Fig. 7:**
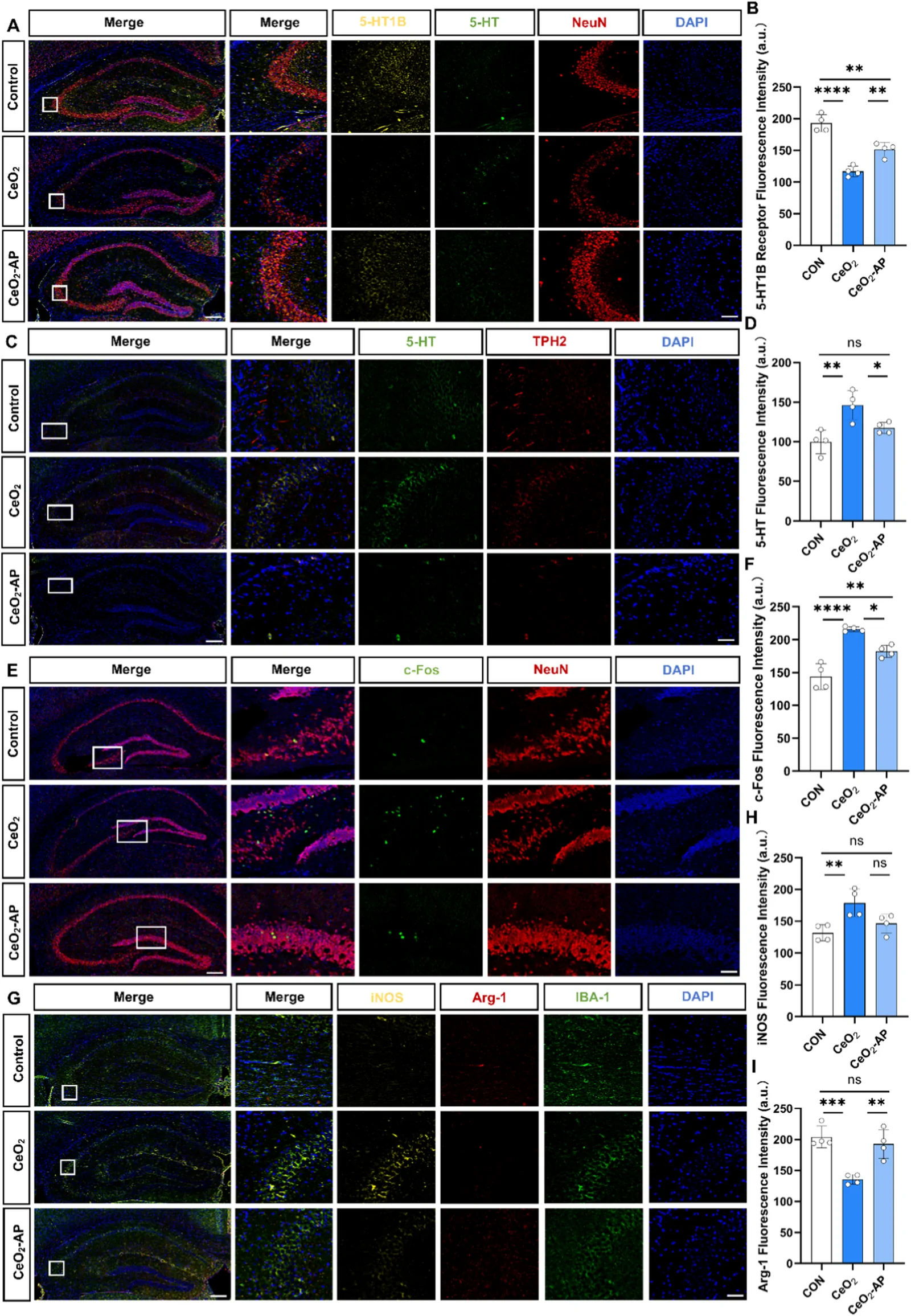
AP ameliorates CeO_2_ NR-induced serotonergic dysregulation, neuronal hyperactivation, and neuroinflammation. (A, B) Immunofluorescence staining and quantitative analysis of the 5-HT1B receptor. Scale bar: 100 μm. Scale bar for enlarged images: 25 μm (n = 4). (C, D) Immunofluorescence and analysis of 5-HT. Scale bar: 100 μm. Scale bar for enlarged images: 30 μm (n = 4). (E, F) Immunofluorescence staining and quantitative analysis of c-Fos. Scale bar: 100 μm. Scale bar for enlarged images: 30 μm (n = 4). (G-I) Immunofluorescence staining and quantitative analysis of iNOS and Arg-1. Scale bar: 100 μm. Scale bar for enlarged images: 25 μm (n = 4).

Regarding the reversal of CeO_2_-induced 5-HT1B receptor downregulation by anpirtoline treatment, our primary hypothesis is that anpirtoline exerts a protective/salvage effect that prevents further reduction of 5-HT1B receptor expression. In mice not treated with anpirtoline, the high-dose toxicity of CeO_2_ leads to exacerbated disruption of 5-HT metabolism, accompanied by an inflammatory microenvironment and neuronal hyperexcitability. Under the combined influence of these factors, 5-HT1B receptor expression is further suppressed in untreated mice. In contrast, anpirtoline exerts a salvaging effect that alleviates the disturbances in inflammation and 5-HT metabolism, thereby preventing further decline in 5-HT1B receptor expression. In addition, anpirtoline may protect the receptor from degradation through ligand occupancy, thereby increasing detectable receptor protein levels. In certain G protein-coupled receptors, agonists can increase the number of receptor binding sites through a mechanism of “ligand-induced protection from receptor instability,” independent of signal transduction [38].

It should be noted that the neurological abnormalities induced by CeO_2_ NRs exposure are likely the result of multiple mechanisms acting in concert. First, the 5-HT1B receptor not only functions as an autoreceptor regulating 5-HT release but also exerts inhibitory regulation over glutamate and gamma-aminobutyric acid (GABA) release [39]. Moreover, previous studies have demonstrated that CeO_2_ can induce inflammatory responses in brain tissue. Our observed high-dose CeO_2_ NRs exposure may simultaneously affect the nervous system through the following pathways. On one hand, 5-HT1B receptor downregulation leads to serotonergic dysregulation, on the other hand, it indirectly affects neuronal function by inducing an inflammatory microenvironment. Either of these mechanisms could contribute to neuronal hyperexcitability and the abnormal exploratory behaviors observed in mice.

Previous studies showed that CeO_2_ induce neurotoxicity, neuronal death and oxidative stress in mouse brain via intraperitoneal and intranasal exposure [21], [23], [24]. Our in vitro data also confirmed high-dose CeO_2_-induced neuronal death, and in vivo data showed brain inflammation. However, unlike previous high-dose toxicity studies, we used a lower in vivo dose with multi-omics to investigate neurotransmitter, excitability and behavioral changes, rather than simply assessing cell death. There are conflicting reports in the literature regarding the biological effects of CeO_2_, with some studies demonstrating its antioxidant properties and others showing that it induces oxidative stress in multiple organs. This is mainly attributable to differences in dosage: at low concentrations, the Ce^3+^/Ce^4+^ redox cycle operates efficiently to scavenge ROS. At high concentrations, this effect reverses, and CeO_2_ exhibit pro-oxidant and cytotoxic effects. The route of exposure significantly affects toxicity outcomes. In current studies, intranasal administration frequently induces brain toxicity, whereas oral and intraperitoneal administration lead to multi-organ distribution and oxidative stress [40]. This indicates that different routes of CeO_2_ entry into the body produce distinct toxic effects on different organs.

Nevertheless, this study still has some limitations. Regarding the translation of animal doses to human exposure levels, significant species differences exist in the nose-to-brain transport of nanomaterials. Mice differ substantially from humans in nasal cavity anatomy and olfactory epithelium proportion, resulting in a much higher olfactory deposition dose per unit surface area. This implies that the doses used in mouse studies cannot be directly extrapolated to human exposure levels.

## 4 Conclusion

This study explored the possible mechanisms of neurological injury caused by short-term intranasal delivery of CeO_2_ NRs. Based on the above results, these CeO_2_ NRs are able to reached and accumulated in the hippocampus and striatum. CeO_2_ NRs exposure caused abnormal exploratory behaviors in the animals, epileptiform changes in the EEG, and increased sensitivity to PTZ-induced seizures. Integrated multi-omics and histological analyses further revealed that CeO_2_ NRs may alter neural network excitability by downregulating *Htr1b* expression, disrupting 5-HT homeostasis, promoting neuroinflammation and neuronal hyperexcitability. Pharmacological activation of the 5-HT1B receptor by AP reversed the behavioral, electrophysiological and molecular abnormalities induced by CeO_2_ NRs, and thus impaired 5-HT1B receptor signaling was considered as a key contributing mechanism of this neurotoxicity. In short, the experiments have provided some support for enhancing the safety assessment of CeO_2_-based nanomaterials and put forward some strategies to improve their potential neurotoxic effects.

## CRediT authorship contribution statement

**Mingshan Liu:** Writing – original draft, Methodology, Formal analysis, Data curation, Conceptualization. **Yuedong Li:** Writing – original draft, Methodology, Formal analysis, Data curation, Conceptualization. **Chunhao Shen:** Writing – original draft, Methodology, Formal analysis, Data curation, Conceptualization. **Hongyi Huang:** Data curation, Conceptualization. **Quanlei Liu:** Data curation, Conceptualization. **Bin Fu:** Methodology. **Bihan Wu:** Methodology. **Xiaomin Wu:** Methodology. **Yumin Luo:** Writing – review & editing, Supervision. **Yongzhi Shan:** Writing – review & editing, Supervision. **Xuegang Niu:** Writing – review & editing, Supervision. **Guoguang Zhao:** Writing – review & editing, Supervision, Resources, Project administration.

## Publication consent

Publication consent has been obtained from the participants.

## Ethics approval and consent to participate

All animal experiments were approved by the Institutional Animal Care and Use Committee of Xuanwu Hospital, Capital Medical University (Approval No. XW-20230605-1).

## Funding

The Beijing Natural Science Foundation-Daxing Innovation Joint Fund (L246017). The Natural Science Foundation of Fujian Province (No. 2025J08169). The Beijing Municipal Science &amp; Technology Commission (Z221100007422016)

## Declaration of competing interest

The authors declare that they have no known competing financial interests or personal relationships that could have appeared to influence the work reported in this paper.

## Data Availability

Data will be made available on request.
