# Supplementary material for: High-dose cerium dioxide nanorods induce behavioral and electroencephalographic abnormalities through 5-HT1B receptor signaling disruption with an evaluation of anpirtoline's therapeutic potential
Source: Mater Today Bio. 2026 Oct 14;41:103749. doi: 10.1016/j.mtbio.2026.103749 (PMC13695163; doi:10.1016/j.mtbio.2026.103749)
Supplement: Supplementary file 1 [file mmc1.pdf]

High-Dose Cerium Dioxide Nanorods Induce Behavioral and Electroencephalographic Abnormalities through 5-HT1B Receptor Signaling Disruption with an Evaluation of Anpirtoline's Therapeutic Potential

Supplementary Figures

Figure S1

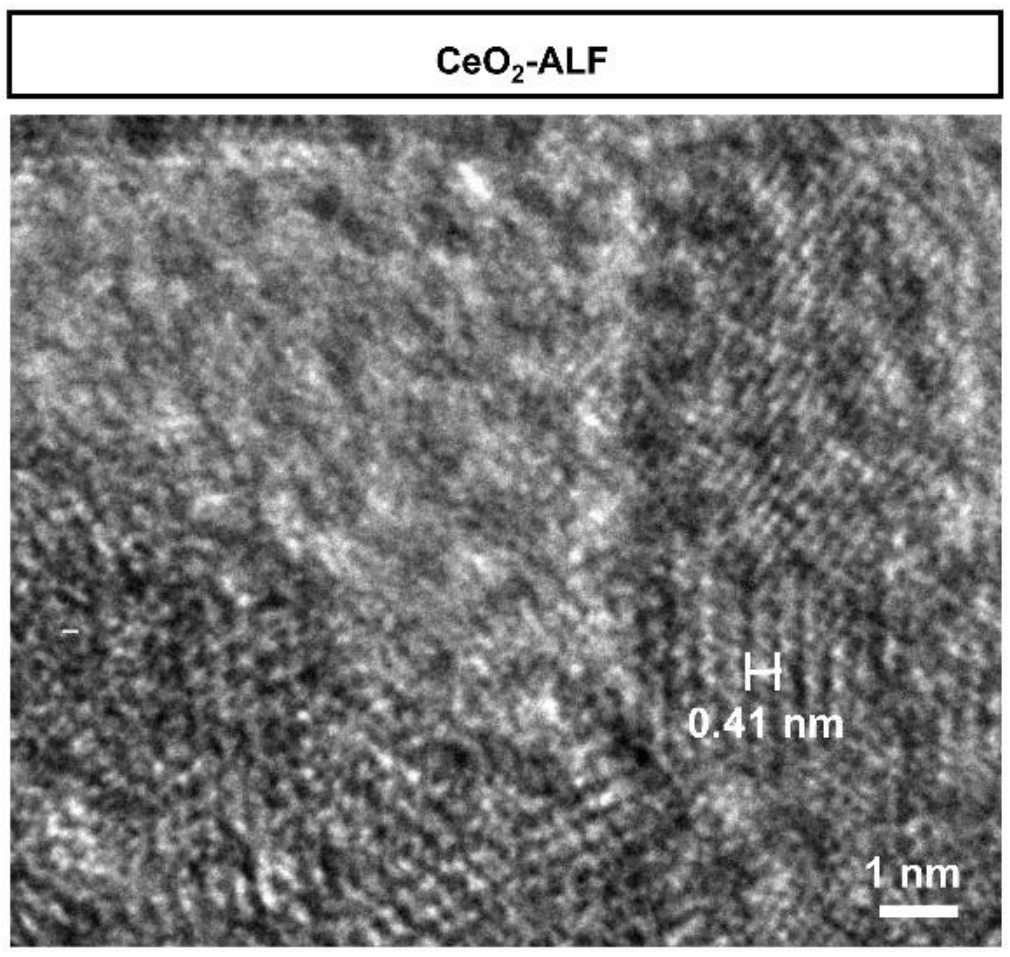

Fig. S1. HRTEM images of CeO_2-ALF NRs.

Figure S2

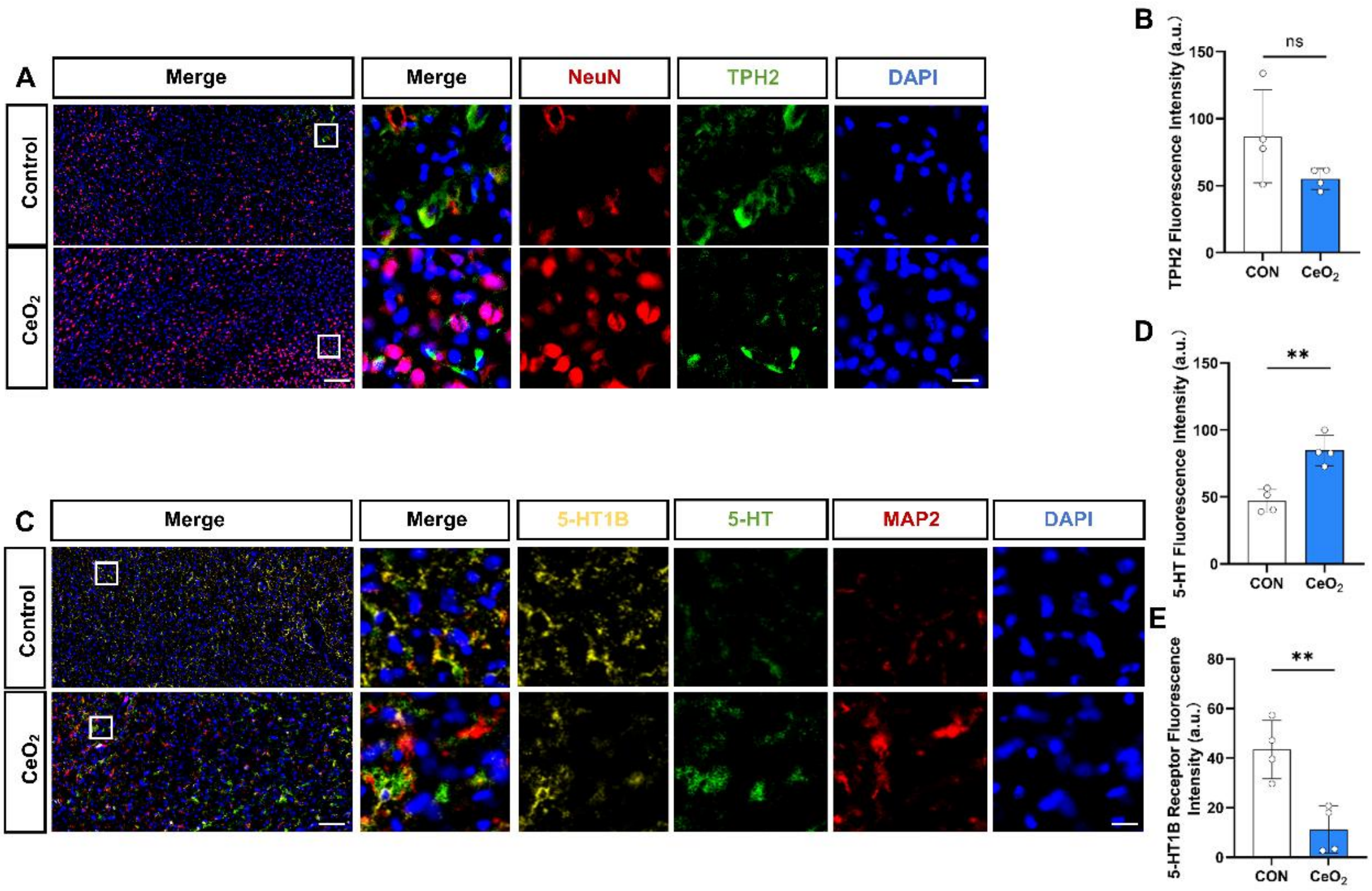

Fig. S2. CeO_2 NRs exposure alters 5-HT-related markers in the striatum. (A, B) Immunofluorescence staining and quantitative analysis of TPH2. Scale bar: 100 µm. Scale bar for enlarged images: 25 µm (n = 4). (C-E) Immunofluorescence staining and quantitative analysis of the 5-HT1B receptor and 5-HT. Scale bar: 100 µm. Scale bar for enlarged images: 25 µm (n = 4).

Figure S3

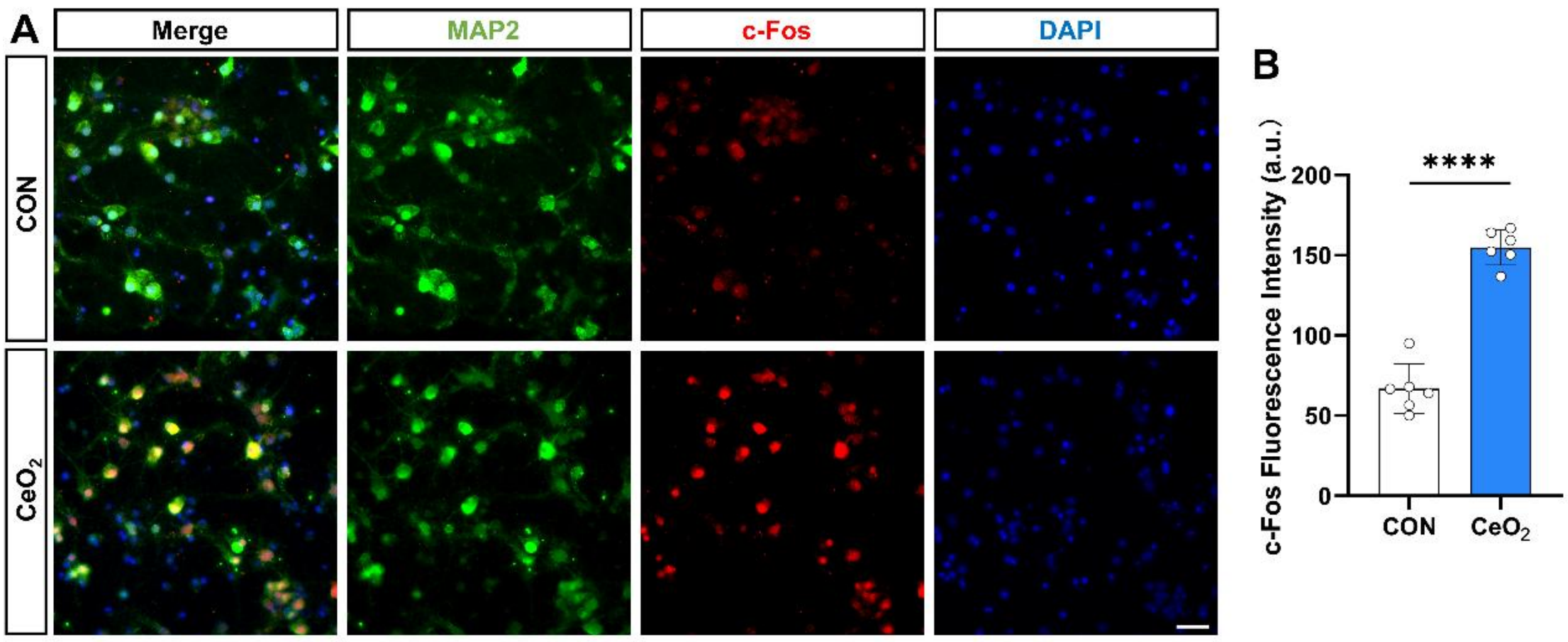

Fig. S3. Immunofluorescence staining for neuronal excitability. (A) Immunofluorescence staining images, scale bar: 20 µm. (B) Statistical analysis of the mean fluorescence intensity of c-Fos (n = 6).

Figure S4

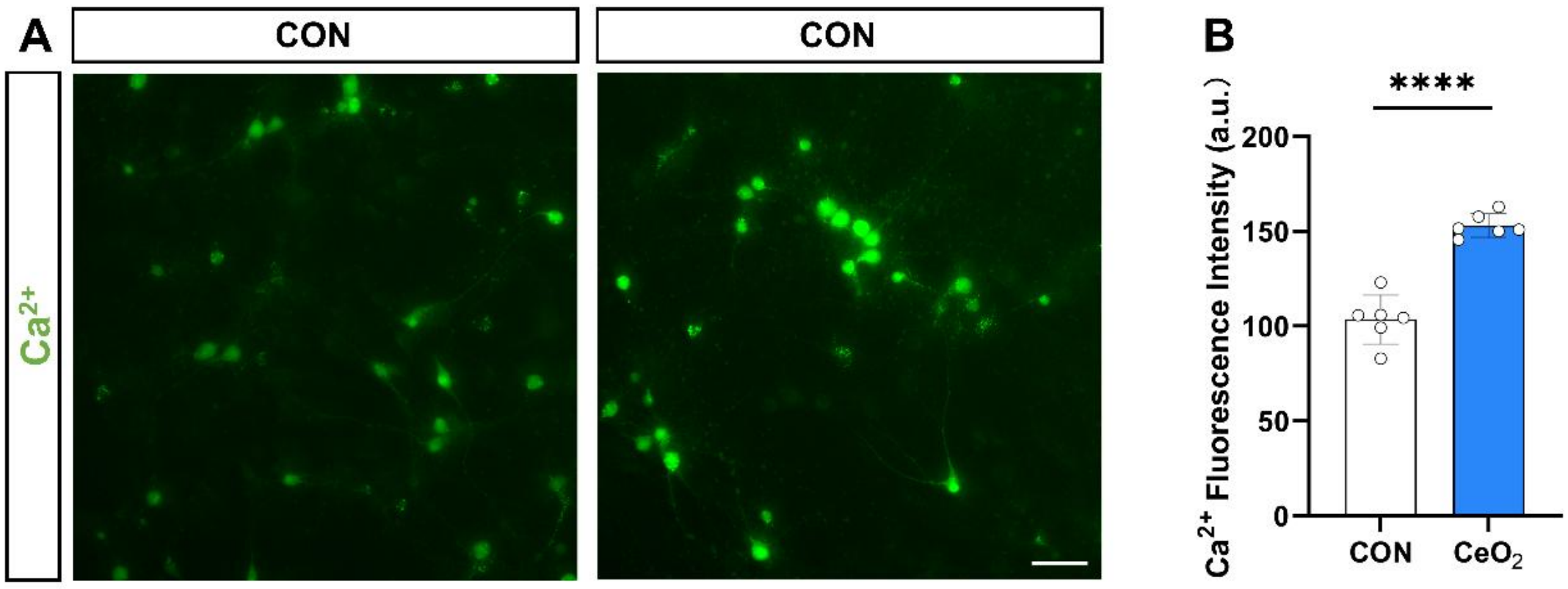

Fig. S4. Detection and analysis of neuronal calcium (Ca^{2+}) content. (A) Immunofluorescence images of neuronal Ca^{2+} content, scale bar: 20 µm. (B) Statistical analysis of the mean fluorescence intensity of Ca^{2+} (n = 6).

Figure S5

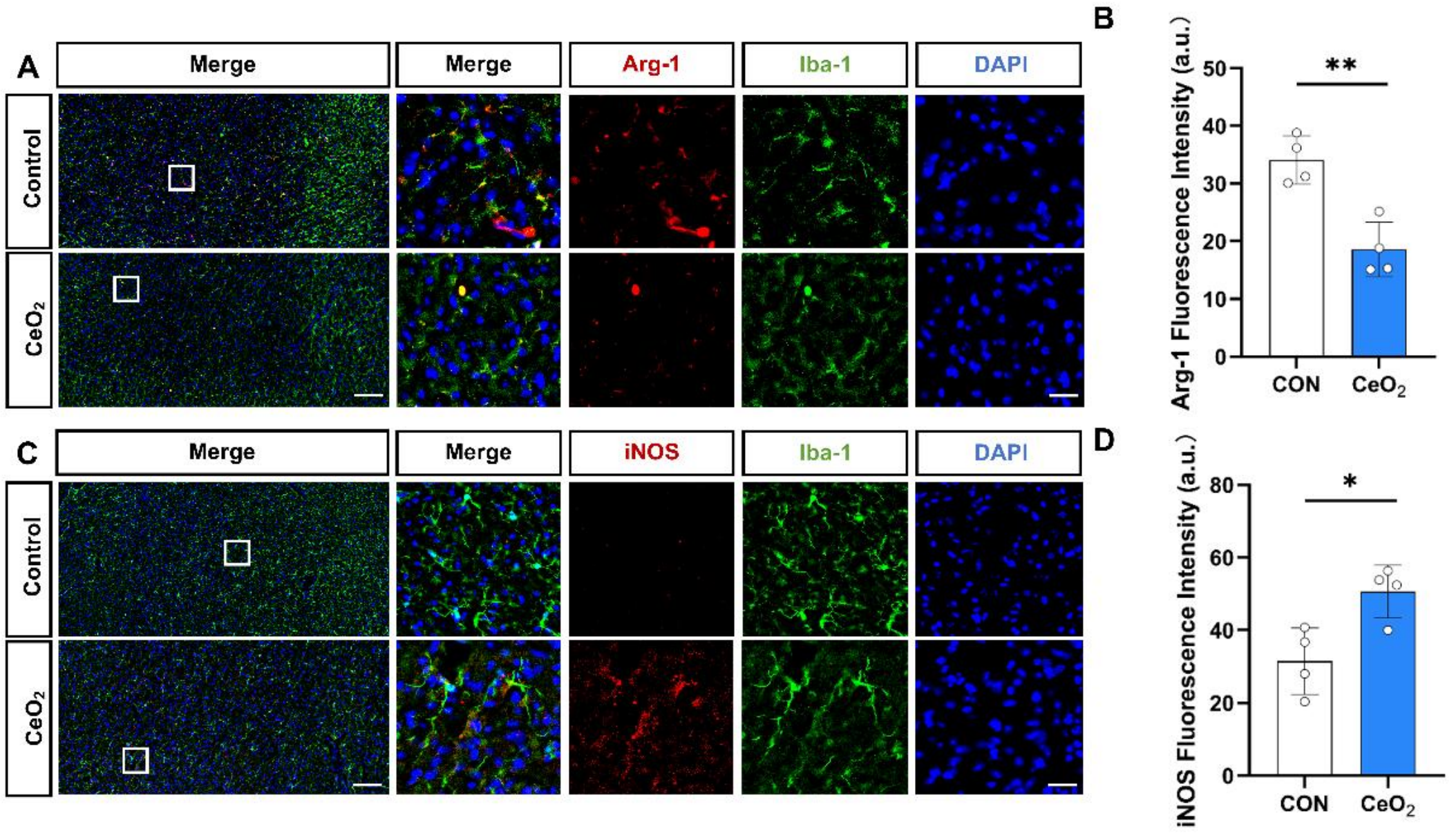

Fig. S5. CeO_2 NRs exposure induces inflammatory microenvironmental alterations in the striatum. (A, B) Immunofluorescence staining and quantitative analysis of Arg-1. Scale bar: 100 µm. Scale bar for enlarged images: 25 µm (n = 4). (C, D) Immunofluorescence staining and quantitative analysis of iNOS. Scale bar: 100 µm. Scale bar for enlarged images: 25 µm (n = 4).

Figure S6

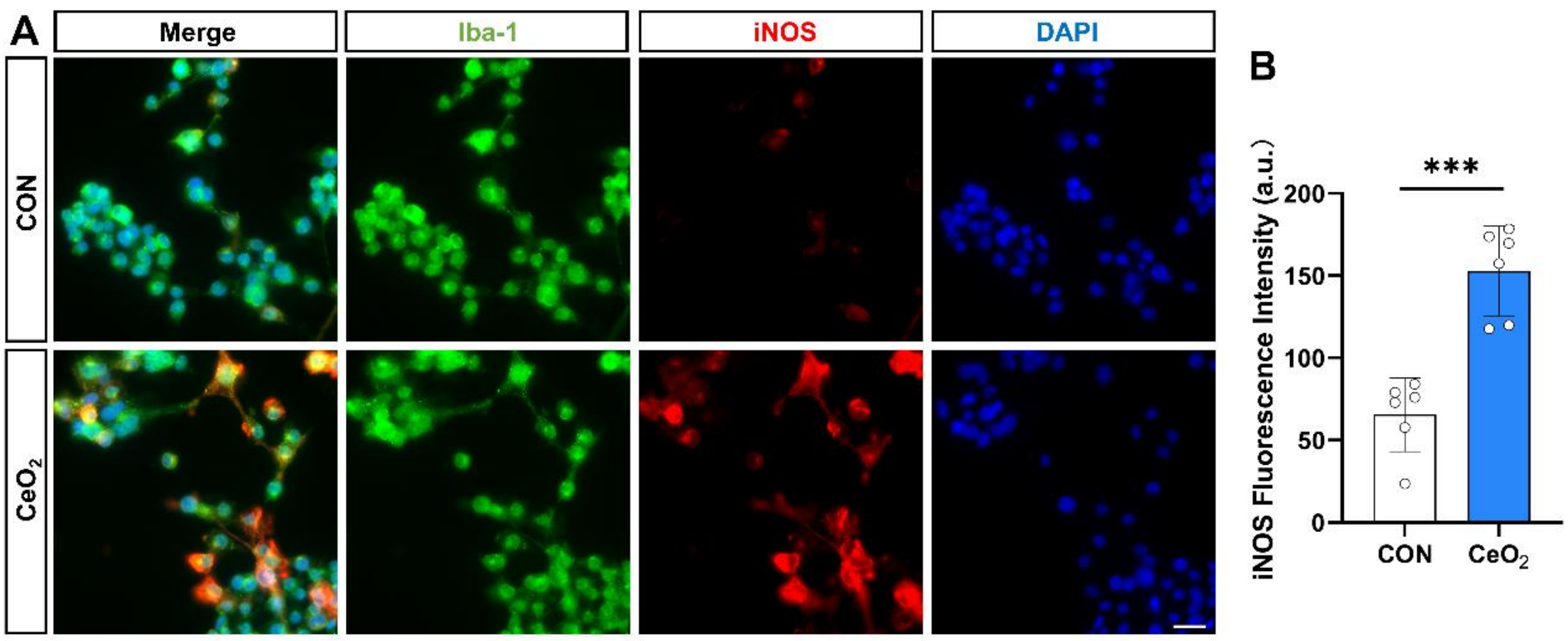

Fig. S6. Immunofluorescence staining and analysis for inflammation in BV2 cells. (A) Immunofluorescence staining images, scale bar: 20 µm. (B) Statistical analysis of the mean fluorescence intensity of iNOS (n = 6).

Figure S7

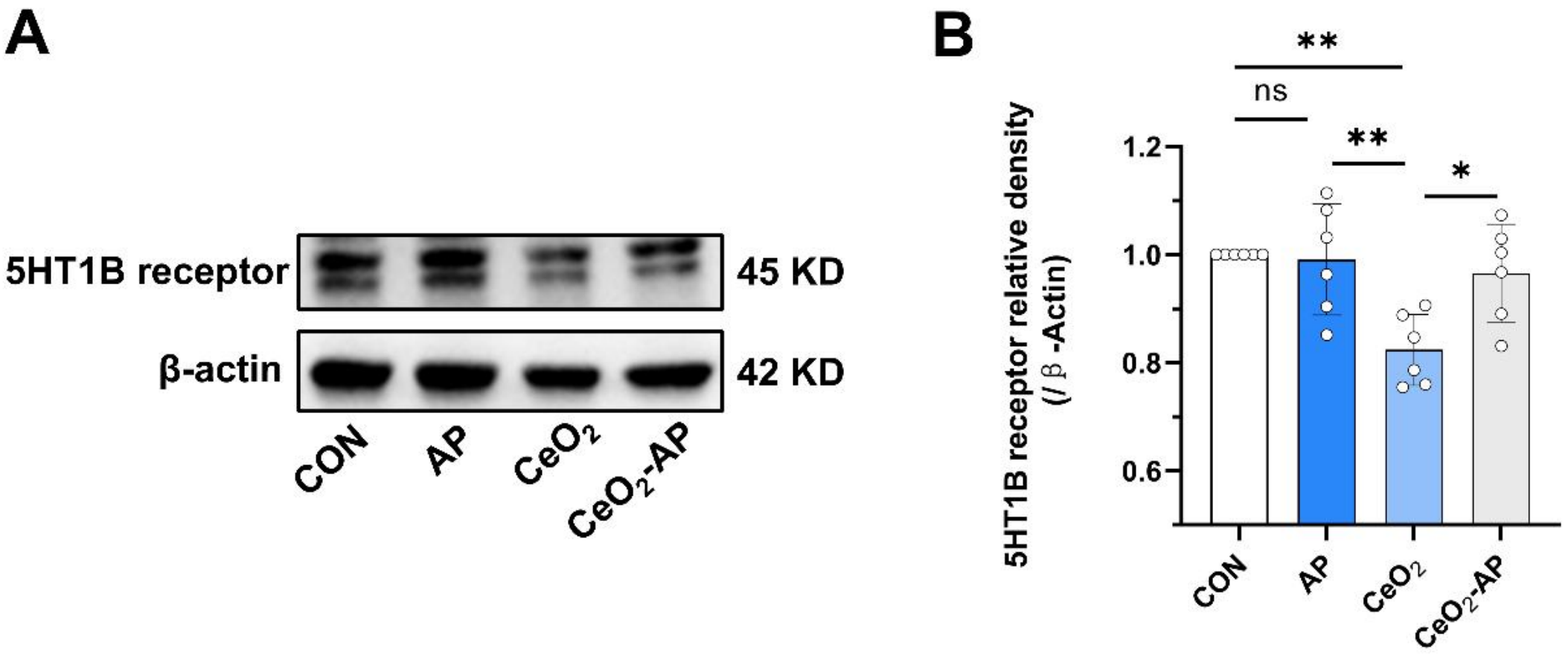

Fig. S7. Image analysis of Western blot for 5-HT1B receptor expression levels. (A) Western blot bands of 5-HT1B receptor. (B) Quantitative normalization analysis of 5-HT1B receptor expression levels (n = 6).

Figure S8

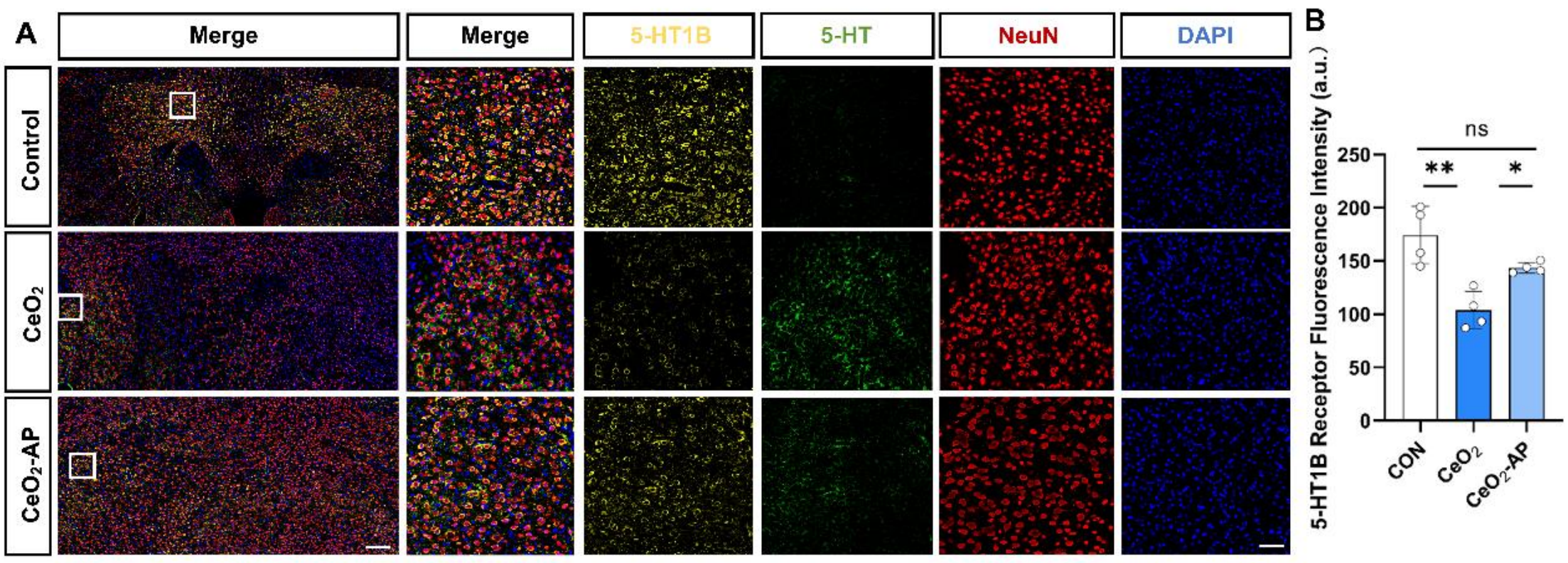

Fig. S8. AP upregulates 5-HT1B receptor expression in the striatum. (A, B) Immunofluorescence staining and quantitative analysis of the 5-HT1B receptor. Scale bar: 100 μm. Scale bar for enlarged images: 25 μm (n = 4).

Figure S9

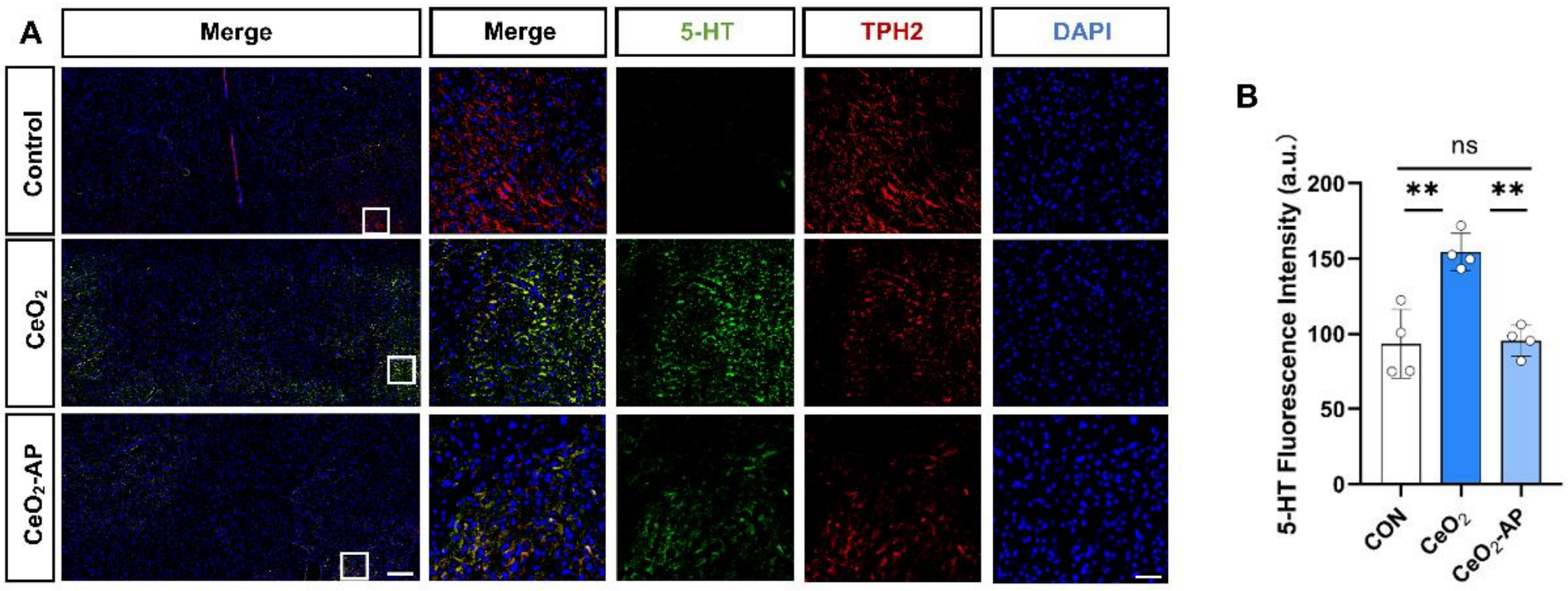

Fig. S9. AP ameliorates 5-HT dysregulation in the striatum. (A, B) Immunofluorescence staining and quantitative analysis of 5-HT. Scale bar: 100 μm. Scale bar for enlarged images: 25 μm (n = 4).

Figure S10

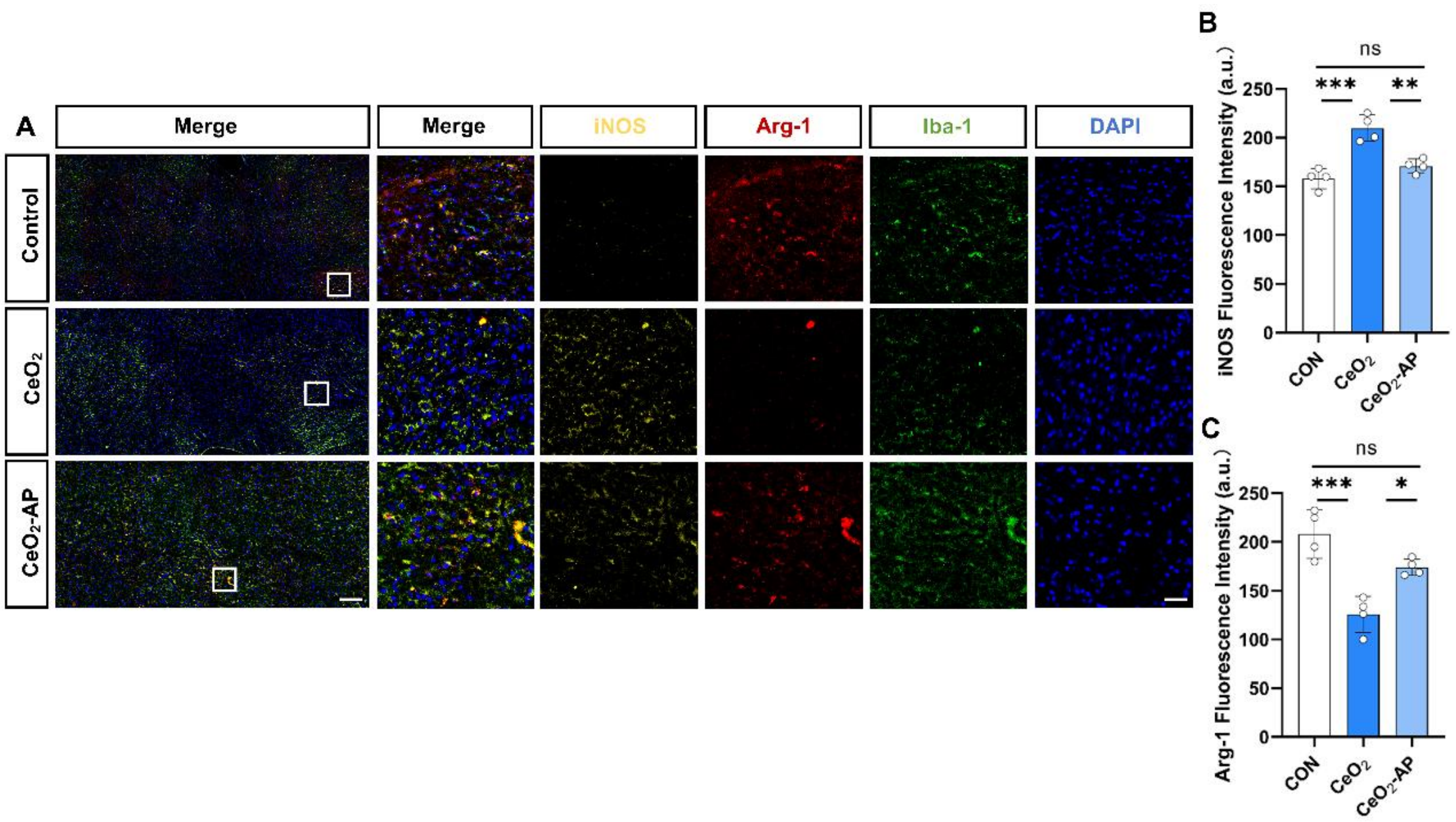

Fig. S10. AP ameliorates inflammatory microenvironmental alterations in the striatum. (A-C) Immunofluorescence staining and quantitative analysis of iNOS and Arg-1. Scale bar: 100 μm. Scale bar for enlarged images: 25 μm (n = 4).
